# Spatial Diversity of Marine Protists Along the Northern Norway Fjords During the Sub‐Arctic Summer

**DOI:** 10.1111/jeu.70133

**Published:** 2026-10-10

**Authors:** Sandra Gran‐Stadniczeñko, Konstantinos Anestis, Carina Bunse, Bente Edvardsen, Philipp Egbers, Rohan Henkel, Cora Hörstmann, Kerstin Klemm, Nancy Kühne, Franco Moritz, Stefan Neuhaus, Luka Šupraha, Jan Tebben, Daniela Voss, Sabrina N. Wilms, Sylke Wohlrab, Uwe John

**Affiliations:** ^1^ Norwegian Institute for Water Research (NIVA) Oslo Norway; ^2^ Section for Aquatic Biology and Toxicology, Department of Biosciences University of Oslo Oslo Norway; ^3^ Ecological Chemistry, Department of Biosciences Alfred Wegener Institute (AWI), Helmholtz Centre for Polar and Marine Research Bremerhaven Germany; ^4^ Department of Marine Sciences University of Gothenburg Göteborg Sweden; ^5^ ICBM: Institute for Chemistry and Biology of the Marine Environment University of Oldenburg Oldenburg Germany; ^6^ Aix Marseille University, Université de Toulon, CNRS, IRD, MIO UM 110 Marseille France; ^7^ Analytical BioGeoChemistry Helmholtz Zentrum München, German Research Center for Environmental Health Neuherberg Germany; ^8^ Helmholtz Institute for Functional Marine Biodiversity at the University of Oldenburg (HIFMB) Oldenburg Germany

**Keywords:** biodiversity, metabarcoding, microbial eukaryotes, polar, SSU rRNA gene, summer community, trophic state

## Abstract

Polar coastal waters are highly complex and productive ecosystems that experience increasingly higher average water temperatures, shallower and stronger stratification, and higher glacier‐melting rates. Yet, the environmental complexity and high microbial diversity within fjords pose challenges to understanding the impacts of these changes and the ecological processes driving them. Here, we studied protists and prokaryote communities, their distribution, and functional ecology along the northern Norwegian coast in summer 2019 using SSU rRNA gene metabarcoding. Physicochemical data provided insights into the main drivers for differences in plankton communities. We found significant differences in alpha and beta diversity among the different fjords and between the western (Vesterålen, Balsfjorden, and Lyngenfjord) and northern areas (Porsangerfjord, Laksefjord, and Tanafjord). Temperature, salinity, and phosphate are the main drivers for community composition differences, positively correlating with the western fjords and negatively with the northern ones. Metabarcoding analyses revealed dinoflagellates, cryptophytes, and ochrophytes as the most abundant groups on the north Norwegian coast and confirmed the bloom of the ichthyotoxic haptophyte *Chrysochromulina leadbeateri* in Balsfjorden. Our comprehensive spatial study of communities and key contributors across different fjords set a baseline for future environmental monitoring of these unique and productive ecosystems, which are subject to rapid environmental changes.

## 1 Introduction

Marine coastal areas are among the most productive ecosystems in the world (Muller‐Karger et al. 2005). Marine planktonic protists, unicellular eukaryotes, include primary producers (phytoplankton), heterotrophs, mixotrophs (which are both phototrophic and heterotrophic), and parasites that are essential in sustaining this productivity as well as other ecosystem functioning and biogeochemical processes at large scale (Massana et al. 2011; Sherr and Sherr 2002; Worden et al. 2015). Specifically, within the marine unicellular plankton communities, protists account for about one‐third of the total marine pelagic biomass (Bar‐On et al. 2018), and play a critical role in marine carbon cycling by both fixing carbon dioxide (CO_2_) through autotrophy (oxygenic photosynthesis) and incorporating organic carbon through phagotrophy, thereby influencing the productivity and stability of higher trophic levels and overall ecosystem functioning (Ptacnik et al. 2008; De La Rocha and Passow 2007). While protists are significant contributors to marine carbon cycling, they interact closely with bacteria and some archaea, collectively shaping the base of the marine food web (Worden et al. 2015).

Climate change induces temperature increases about four times faster in polar than temperate regions (Rantanen et al. 2022). As a result, polar (coastal) waters have experienced higher average summer temperatures and stronger, shallower stratification since the late 20th century, accompanied by increased rates of glacier melt relative to pre‐1990 conditions (Rantanen et al. 2022). Additionally, increased water temperatures change the extent and timing of the phytoplankton growth season (Coello‐Camba and Agustí 2017). These environmental changes represent fundamental stressors for aquatic organisms in polar fjords and the adjacent coastal systems. Higher temperatures and freshwater input from increased precipitation and snow and ice melt lead to stronger stratification, less mixing and nutrient exchange with deep waters, and, therefore, to alterations of microbial community structure at the land‐sea interface (Fu et al. 2016; Rhein et al. 2013).

The fjord systems in Northern Norway are influenced by the Norwegian Coastal Current (Wassmann et al. 1996), as well as by both the North Atlantic Current/Norwegian Sea and the Barents Sea water masses. Two regions with different environmental conditions can be defined along the Northern Norwegian coast: (i) the temperate to sub‐polar North‐Western fjords and the Vesterålen archipelago region in the Nordland and Troms counties, which are strongly affected by the Norwegian Sea water, Norwegian Coastal Current, and the Lyngenfjord, the transition area to the Barents Sea (Skjoldal and Saetre 2004); (ii) the polar to sub‐polar northern fjords along the Finnmark coast that have a strong polar influence from the Barents Sea and a warmer water influence from the Norwegian Coastal Current, which follows the coast eastwards toward Novaya Zemlya (Russia) during summer (Sakshaug et al. 2009). Only some Norwegian fjords, such as Porsangerfjord, experience sea ice during winter and spring (Wassmann et al. 1996), whereas others, such as Balsfjord and Malangen, are ice‐free all year‐round (Archer et al. 2000). Further, Norwegian fjords usually have a pronounced landlocked character due to a sill, a generally large depth and length, and intermittent periods of bottom water stagnation, during which reduced circulation can lead to oxygen depletion. The fjords in Northern Norway receive less freshwater input than those in Western and Southern Norway, except for spring and summer, when riverine and snowmelt runoff significantly alter their hydrography (e.g., Svendsen 1995). The most northern fjords, such as Porsangerfjord, Laksefjord, and Tanafjord, are broader at their mouth and have a larger input of cold Barents Sea water than those further south, allowing for greater water exchange with oceanic water (Wassmann et al. 1996).

Hydrodynamic conditions strongly influence the composition and distribution of plankton communities. On a larger scale, plankton populations are transported northwards by the inflow of water from the Norwegian Sea and the Norwegian coastal current, becoming part of the Barents Sea community composition and production system (Hegseth and Sundfjord 2008; Sakshaug et al. 2009). Previous studies of Northern Norwegian fjords, based on microscopically identified taxa, have found a similar phytoplankton species composition among fjords north of 69° N in the Barents Sea (Degerlund and Eilertsen 2010). A characteristic feature of the fjords along the Barents Sea is that spring phytoplankton bloom concentrations are low, and primary production is more spread throughout the whole growth season. This is contrary to what is common further south with pronounced spring bloom, summer recession, and autumn bloom (Eilertsen and Frantzen 2007). The homogeneity of the Atlantic water mass density and the regular wind conditions along the Norwegian Sea coast result in a deep‐water column mixing during winter, followed by a slow stabilization during spring and early summer. Consequently, this results in changes in the growth of photosynthetic protist groups (phytoplankton), ranging from more constrained to more prolonged periods (Behrenfeld et al. 2013; Paulsen et al. 2015). Therefore, studying multiple fjords to enhance the spatial resolution of plankton communities is important, as this will provide a more comprehensive understanding of the region's diverse and dynamic marine ecosystems. Due to the gradual differences in environmental conditions across Northern Norway, this region is a prime location to study the rapid transition of ecosystems in response to climate change, specifically, changes in the diversity of marine microbes in temperate and sub‐Arctic fjords. Yet, phytoplankton does not solely respond to abiotic factors during spring. Together with other organisms such as zooplankton and bacteria, they form interactive communities within the realm of planktonic food webs (Carmack and Wassmann 2006). Bacterial nutrient recycling, competitive interactions, and the release of organic compounds by phytoplankton are among the complex, temporally dynamic interactions between phytoplankton and bacteria (Buchan et al. 2014). Several studies have analyzed the bacterial community composition accompanying spring blooms, and members of bacterial groups such as Flavobacteria, Gammaproteobacteria, and Alphaproteobacteria are typically observed during spring, accompanied by lower species diversity during bloom events (Buchan et al. 2014; Raes et al. 2022). Prokaryotic springtime dynamics additionally show spatial separation between fjords, reflecting variations in environmental parameters such as time and nutrient concentration (Piquet et al. 2016).

Here, we present an extensive spatial study of the coastal microbial community composition, including protists and co‐occurring prokaryotes across different polar fjords in sub‐Arctic areas, using a metabarcoding approach during the cruise HE533 aboard the *R/V* Heincke in 2019. We investigated the Vesterålen archipelago (Lofoten) and five sub‐Arctic fjords: Balsfjord, Lyngenfjord, Porsangerfjord, Laksefjord, and Tanafjord in Northern Norway. During this cruise, massive fish kills took place in Balsfjorden due to blooms of the haptophyte phytoflagellate *C. leadbeateri* (Aalto et al. 2023; John et al. 2022; Otte et al. 2025). The main aim of this study is: (i) to describe the protist community and assess taxonomically the trophic mode distribution in fjords along the Northern Norwegian Coast, (ii) to compare the protist community composition and diversity between fjords in temperate and sub‐Arctic areas, and (iii) to identify possible abiotic and biotic factors that may explain some of the differences in community composition, relative abundances, and potential trophic functions by protists within and among the investigated fjords and their corresponding regions. Our study contributes to understanding and identifying potential environmental drivers and factors that impact plankton structure, and helps better monitor and understand these unique and productive ecosystems, which are subject to rapid environmental changes.

## 2 Materials and Methods

### 2.1 Oceanographic Measurements

Seawater samples and CTD data were collected onboard R/V Heincke (HE533) between 20.05.2019 and 06.06.2019 at six transects along the Northern Norwegian coast: Vesterålen (North‐West of Lofoten in the Nordland County), Balsfjord and Lyngenfjord in the Troms County, Porsangerfjord, Laksefjord, and Tanafjord in Finnmark County (Figure 1).

**FIGURE 1:**
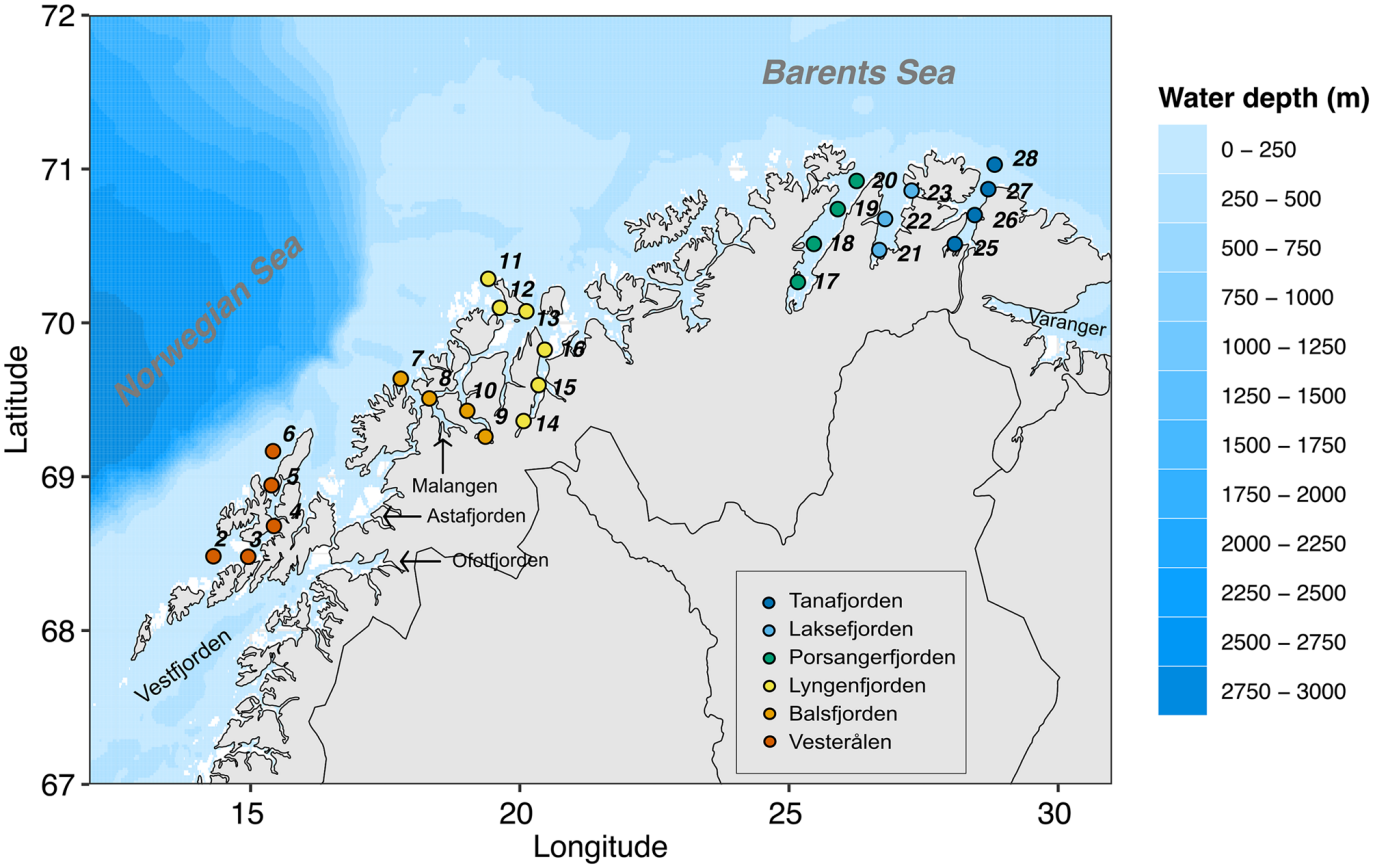
Location of the six transects. Stations 2–6 are in Vesterålen Archipelago, st. 7–10 in Balsfjorden, st. 11–16 in Lyngenfjorden, 17–20 in Porsangerfjorden, 21–23 in Laksefjorden, and 25–28 in Tanafjorden, Northern Norway.

### 2.2 Sampling Plankton and Water

Water column profiling was carried out with a Seabird’ SBE911+’ CTD probe with additional turbidity (WetLabs FLNTU; detection limit: 0.015 μm L^−1^), oxygen (SBE43; initial accuracy: +/− 2% of saturation), and fluorescence (WetLabs FLNTU; detection limit: 0.0015 m^−1^) sensors attached to a water sampling rosette with 10 L Niskin bottles. Before sampling, a CTD cast was performed at each station to measure conductivity, temperature, and pressure along a vertical transect through the water column. Salinity, density, and depth were calculated from these measurements, and the depth of the sub‐surface chlorophyll maxima (SCM, variable per station) was determined. Real‐time CTD data were obtained via the CTD‐client onboard and post‐processed with the software Seasoft V2. The mean value from three depths (3, 15, and 40 m) of each parameter was calculated and used in further analyses (Table S1). The CTD data are available in Pangaea (John and Wisotzki 2019), and detailed post‐processing of the complete CTD workflow is given within the HE533 Data Processing Report (Rohardt 2019).

For nano‐ and picoplankton as well as for bacterial plankton, a total of 24 L of seawater was collected from three depths using Niskin bottles (3 m, SCM, which varied between 8–28 m, and 40 m) with 8 L each and subsequently pooled. A pre‐filtration was performed using gravity filtration over a filter tower set‐up with 200 μm on top and 20 μm mesh sieves below. Twenty liters of the filtrate were further size‐fractioned by in‐line filtrations through 3 μm and 0.2 μm polycarbonate (PC) membrane filters (147 mm diameter, Millipore) with a Sartorius Tripod filtration unit and a peristaltic pump for a maximum duration of 30 min, yielding two size fractions: nanoplankton (20–3 μm) and picoplankton (3–0.2 μm). Filtration of these size fractions was adjusted to as gentle as possible while still allowing the filtration of approx. 10 Liters in time, avoiding changes in temperature and limiting plankton community activity changes. However, sensitivity was always more important than volume, and for each station the volume was noted. A microplankton size fraction (200–20 μm) was obtained from 60 L collected at the same depth as described above using a hose and a membrane pump (QBY‐25 SS; AGB‐Pumpen, Germany), prefiltered with gravity filtration through 200 μm and collected on a 20 μm mesh sieve. Retentates from the filter tower and membrane filters were backwashed with 0.2 μm‐filtered seawater, adjusted to a total volume of 45 mL, and split into three aliquots of 15 mL. Cell suspensions were centrifuged for 15 min at 3600 g at 4°C, and the pellets were resuspended with 700 μL DNA lysis buffer. The 147 mm filters from the smaller cell fractions were cut into equal quarters for distinct purposes (e.g., DNA extraction, RNA extraction, metabolomic analyses). For DNA extraction, one filter quarter from each filter size was folded and placed carefully into vials with glass beads, covered with 700 μL SL1 buffer from the NucleoSpin Soil kit (Macherey‐Nagel, Düren, Germany), vortexed, and frozen at −20°C until further processing.

### 2.3 Nutrient Analyses

Samples for inorganic nutrient analyses (nitrate, nitrite, ammonium, phosphate, and silicate) were collected directly from the Niskin bottles at three depths (3, 15, 40 m) per station and immediately stored at −20°C. Nutrient concentrations were determined with a continuous‐flow autoanalyzer (Evolution III, Alliance Instruments, France) using standard seawater analytical methods for determination of concentrations of nitrate and nitrite (Armstrong et al. 1967), ammonium (Koroleff 1969), silicate (Grasshoff et al. 1999), and soluble reactive phosphate (Eberlein and Kattner 1987) (Table S2; Figures S1, S2). For each chemical parameter and station, a depth‐averaged concentration was calculated using the trapezoidal method. This approach computes the area under the concentration‐depth curve for each interval between the three sampling depths, sums these areas, and divides by the total depth range to obtain a depth‐weighted average concentration (mmol m^−3^; Table S1). This method was preferred to ensure comparability with the DNA samples, which were collected over the same 0–40 m water column.

### 2.4 Metabarcoding of Eukaryotic and Prokaryotic SSU rRNA Genes

DNA was extracted using the NucleoSpin Soil kit (Macherey‐Nagel, Germany) following the manufacturer's protocol. For the cell lysis step, a bead beater (MagNA Lyser, Roche) was used to break up the cells. PCR amplification and library construction were performed as described in Illumina's 16S rRNA gene metabarcoding sequencing library preparation (https://support.illumina.com/downloads/ 16s_metagenomic_sequencing_library_preparation.html, doc.no:15044223B), with slight modifications for 18S rRNA gene amplicon preparation. The prokaryotic community composition was determined using primers targeting the V4 hypervariable region (515F—806R) (Weydmann‐Zwolicka et al. 2024), and eukaryotic DNA was amplified by targeting the V4 18S rRNA gene using the primers TA‐Reuk454FWD1—TAReukREV3 (Stoeck et al. 2010) with overhanging Illumina adapters. Briefly, the 25‐μl PCR reaction mix consisted of 2.5 μL of genomic DNA (5 ng μl^−1^), 5 μL of 1 μM Next. Rev. primer and 12.5 μL of 2× KAPA HiFi HotStart ReadyMix (KAPABiosystems, Boston, US). The PCR program included an initial denaturation at 95°C for 3 min, followed by 25 cycles of denaturation at 95°C for 30 s, annealing at 55°C for 30 s, extension at 72°C for 30 s, and a final extension at 72°C for 5 min. PCR reactions were cleaned with AMPure XP beads (Beckman Coulter Life Sciences, Indianapolis, US), validated with the 2100 Bioanalyzer (Agilent), and pooled in equimolar concentrations. The amplicon libraries were sequenced using the MiSeq Reagent Kit v3 (600‐cycle) MS‐102‐3003 in a MiSeq Sequencer (Illumina) at the AWI. Raw metabarcoding sequences are publicly available in the European Nucleotide Archive (ENA) under the accession number ERP134612 (https://www.ebi.ac.uk/ena/browser/view/ERP134612).

### 2.5 Bioinformatic Processing

Primers and spurious sequences were trimmed with CUTADAPT 2.8 (Martin 2011). The DADA2 pipeline (Callahan et al. 2016) was used to quality‐filter, trim, remove chimeras, and denoise reads into amplicon sequence variants (ASVs). The taxonomic assignment was performed against the Protist Ribosomal Reference Database (PR2 v.4.12, Guillou et al. 2013). In total, 5150 ASVs were generated and taxonomically annotated at least to the phylum level by DADA2 and PR2 and phylogenetic placement (Šupraha et al. 2022). To assign ASVs to trophic modes (autotrophic, heterotrophic, mixotrophic), each ASV was first matched to the HELCOM dataset (Olenina et al. 2006) and to the functional traits table compiled by Schneider et al. (2020), both of which provide trophic mode information at the species level. ASVs that lacked species‐level matches in these datasets were subsequently checked against the PR2 classification, which assigns trophic modes at the class level (Guillou et al. 2013). Dinoflagellate ASVs that could not be confidently assigned to phototrophic, mixotrophic, or heterotrophic categories based on the available literature were retained as dinoflagellates_UT (unknown trophic mode). After this second classification, some non‐dinoflagellate ASVs were still not assigned and were left as Unknown Trophy.

### 2.6 Statistical Analyses

Statistical analyses and plots were performed in R software v.3.6.1 (R Core Team 2020) using multiple R packages. The station map was generated with custom‐adapted versions of the ‘get.bathymetry’ and ‘plot. bathymetry’ functions (https://github.com/markolipka/ggPlotBathy/blob/master/ggPlotBathymetry.R). Treemap plots representing the entire Northern Norway protist community composition were produced based on read abundance and ASV richness using the ‘geom_treemap’ option in the ggplot2 package (Wickham 2016). The dataset was rarefied to even sample sizes using the ‘rarefy_even_depth' function in the phyloseq package (McMurdie and Holmes 2013) to compare communities among stations and fjords. Due to large read‐number differences between size fractions, rarefaction was carried out on separate size‐fraction datasets to their minimum sample size (6974 picoplankton, 48,970 nanoplankton, 6893 microplankton), after which all size classes were merged into a single dataset for comprehensive analysis. The ‘plot‐anova‐diversity’ function in the microbiomSeq (Ssekagiri 2020) package was used to calculate alpha‐diversity community metrics (Shannon, Evenness, Richness), perform pairwise ANOVA between stations and fjords, and generate boxplots of the results with annotations at a 0.05 significance level. The unique and shared ASVs at each fjord were identified by creating and visualizing a fjord intersection matrix with the ‘upset’ function in the UpSetR package (Conway et al. 2017).

To obtain beta‐diversity matrices, the community data was first Hellinger‐transformed as recommended for the ordination of species abundance data (Legendre and Gallagher 2001) with the ‘decostand’ function, and ‘vegdist’ was subsequently used to compute Bray‐Curtis dissimilarities (Bray and Curtis 1957) in the vegan package (Oksanen et al. 2020). The relationship between protist community composition and environmental variables was explored using the distance‐based Redundancy Analysis (db‐RDA) with the ‘dbrda’ function and the Permutational Multivariate Analysis of Variance (PERMANOVA) with the ‘adonis2’ function in the vegan package (Oksanen et al. 2020). A db‐RDA significance model was tested using permutational analysis for the overall model and the significance of individual factors, as in Table S1, following the code published by Juottonen et al. (2020). Distance‐based redundancy analyses were then performed on the relative proportions to determine how the significant factors correlate with the taxonomic divisions, the ten most abundant ASVs, and the different trophic modes.

### 2.7 Artificial Intelligence Tool

Grammarly (Grammarly Inc.) was used for grammar and language editing of this manuscript. All scientific content is the result of the authors' work alone.

## 3 Results

### 3.1 Spatial Variations of Physicochemical Factors

Water temperatures (average of 3, 15, and 40 m depth) varied among stations from 2.85°C to 6.55°C (mean 5.01°C; Figure 2) and were warmer in the more western fjords (Table S1). Also, transect plots showed the water column at the inner part of the fjords to be colder than at the outer stations (Figure S1). Salinity varied slightly among stations (32.1–34.3, mean 34 PSU), with the lowest registered at st09, inner Balsfjord (Figure S1). The average integrated phosphate concentration was 0.33 μM and varied among stations, with the lowest values found at st05 (Vesterålen, 0.12 μM) and the highest at st16 (Lyngenfjord, 0.54 μM). Nitrate (NO_3_) positively correlated with phosphate (PO_4_), silicate (SiO_4_), and nitrite (NO_2_). NO_2_ correlated positively with PO_4_ and ammonium (NH_4_), whereas temperature, PO_4_, NO_2,_ and NO_3_ correlated negatively with oxygen concentration (ANOVA, *p* < 0.05, Figure S4). Chlorophyll *a* (chl‐*a*) values at SCM (chlorophyll maximum depth) varied between 0.4 and 4.5 μg L^−1^ across all stations except at st10 (Balsfjord), where the concentration reached 17.4 μg L^−1^. Detailed nutrient and chl‐a distributions are shown in Figures S2 and S3 and in Table S2 for all depths.

**FIGURE 2:**
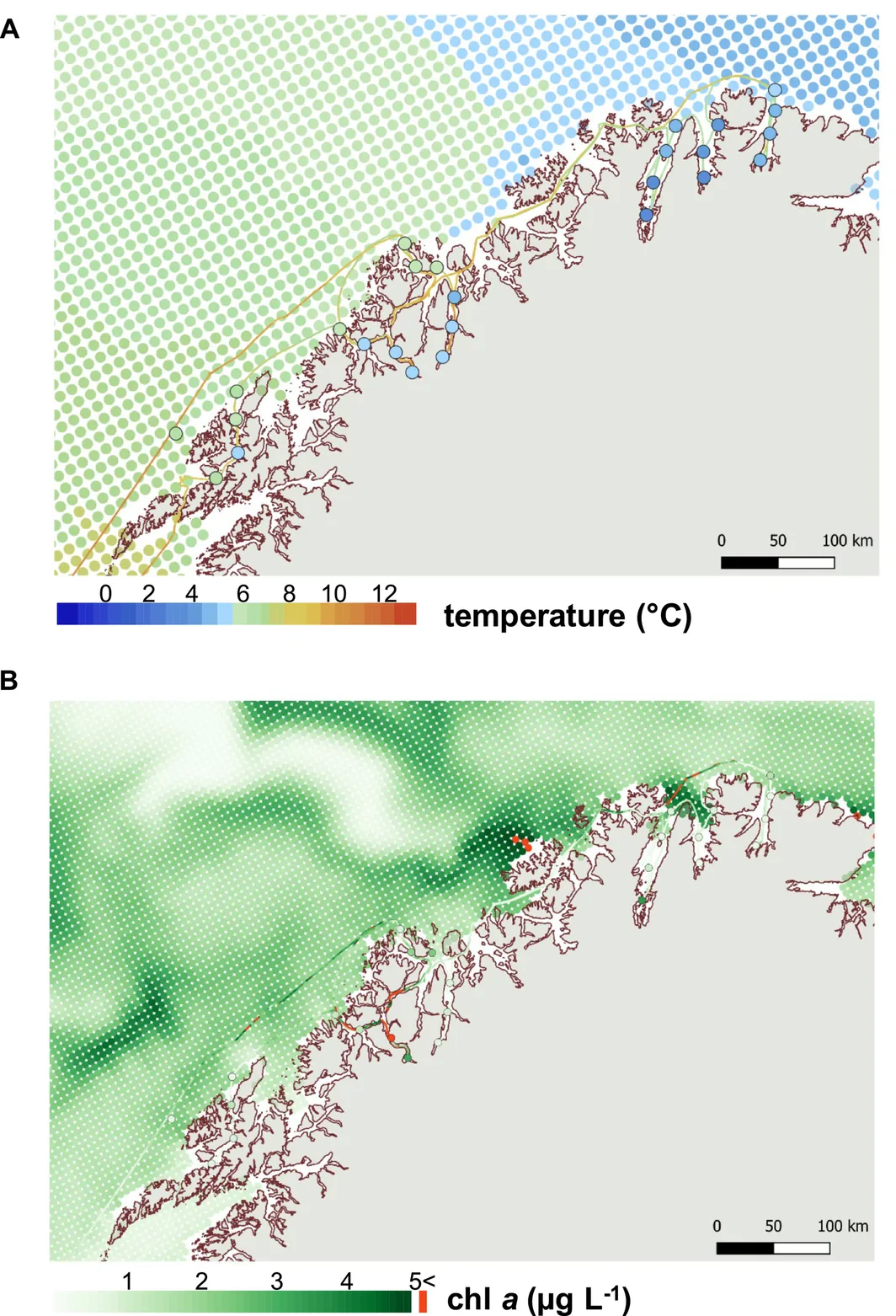
Environmental conditions based on satellite data averaged over sampling expedition and ship‐board measurements (CTD + Ferrybox). (A) sea surface temperature and (B) chlorophyll‐*a* concentrations. Encircled Dot colors in A and B indicate the temperature (A) and chlorophyll‐*a* (B) data from the CTD, and circles without contour indicate data from Ferrybox at each station. Sea surface temperature and chlorophyll‐*a* were obtained by the Copernicus satellite (https://marine.copernicus.eu/) and plotted in gGIS v3.24.

### 3.2 Northern Norway Protist Community Composition

Sequencing and initial quality filtration with the DADA2 pipeline resulted in a total of 13,907,302 18S rRNA gene reads (5150 ASVs, Table S3) with an average fragment length of 383 bp, ranging between 245 and 459 bp. A second manual filtration was performed, consisting of removing all Metazoa, higher fungi (Basidiomycota, Ascomycota), and Viridiplantae (Embryophyceae), as well as sequences identified as nucleomorph genes of cryptophytes, bacteria, and chimeras. This filtering step resulted in 9,547,978 reads corresponding to 4231 ASVs (Table S3).

In the total ASV dataset, 126 ASVs were identified as abundant, each contributing more than 0.1% to the total sequence reads. Collectively, these abundant ASVs account for 83% of the total reads. In contrast to the abundant ASVs, 4016 ASVs each contributed less than 0.05% of the total reads, collectively accounting for only 10.7% of the dataset. The remaining 6.3% of the reads were composed of ASVs that each contributed between 0.05% and 0.1% of the total reads. Most (97.6%) of the ASVs could be taxonomically assigned to at least the Phylum level (Figure S5). The remaining 2.4% were assigned as unclassified eukaryotes. The infrakingdom Alveolata contributed most to ASV richness (40.9% of ASVs), followed by Stramenopiles (24.5%) and Rhizaria (10.3%). Hacrobia was the most abundant group in terms of read numbers (31.1% of total reads), followed by Alveolates (28.8%) and Stramenopiles (19.6%). Archaeplastida, Opisthokonta, Amoebozoa, Apusozoa, and Excavata accounted for less than 10% each of the total ASVs and read abundance. To compare reads among samples, rarefaction was performed to the minimum sample size for each size class (6974 for picoplankton, 48,970 for nanoplankton, and 6893 for microplankton). Subsampling produced 1,633,762 reads assigned to 3441 ASVs (Table S3).

All three alpha diversity indices (richness S, Shannon diversity H′, and Pielou's evenness J') were higher in the Northern area than the western (S_northern_ = 1043, S_western_ = 977; H_northern_ = 4.57, H_western_ = 4.18; J_northern_ = 0.66, J_western_ = 0.61), but only H′ and J' were found to be significantly different (ANOVA *p* ≤ 0.05; Figure 3A). Comparison among fjords showed significant differences in richness and evenness between western and northern fjords (Figure 3A) and between fjords in different areas (Figure 3B). However, Shannon diversity was not significantly different among fjords in the western region.

**FIGURE 3:**
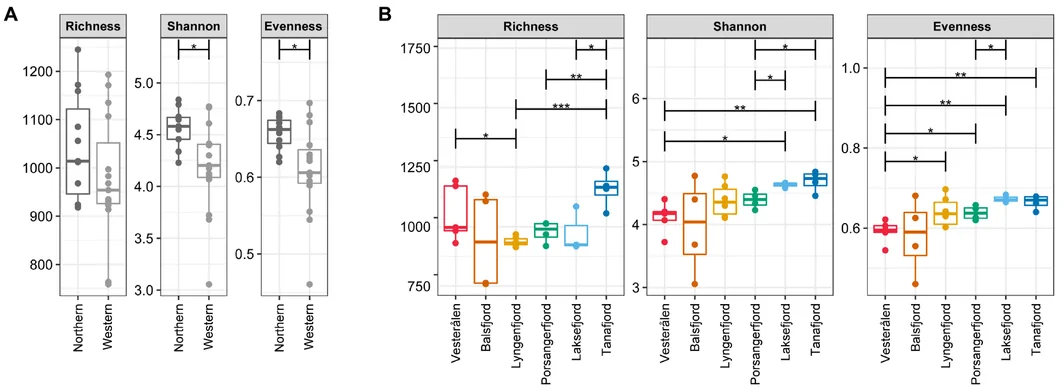
Alpha diversity boxplots for Richness (S), Shannon diversity (H′), and Pielou's evenness (J') indices at the different studied regions, (A) northern and western samples, and (B) the six different fjords. Thick lines indicate average diversity. ANOVA was used to compare diversity across different regions and fjords. Only significant differences are presented: ‘*’ (*p* ≤ 0.05), ‘**’ (*p* ≤ 0.01), and ‘***’ (*p* ≤ 0.001).

### 3.3 Community Composition and Environmental Drivers in Six Regions

All 18S rRNA samples within the same fjords clustered together except for Balsfjord, where samples were widely spread in the NMDS plot (Figure 4), indicating different community compositions. The db‐RDA model showed two environmental factors significantly correlated with the difference in community composition (*p* < 0.005) and accounted for 28% of the total variation: temperature explained the 18.3% (F = 6.12, *p* = 0.001) and salinity the 9.6% (F = 3.19, *p* = 0.004) of variation. The first axis (dbRDA1) explained 66% of the variation (F = 5.89, *p* = 0.001) and was negatively correlated with temperature and salinity. dbRDA2 explained 33.9% of the variation (F = 3.03, *p* = 0.002) and was positively correlated with temperature and negatively correlated with salinity. Temperature and salinity significantly contributed to differences in community composition in Northern Norwegian fjords (*p* < 0.05, Table S4).

**FIGURE 4:**
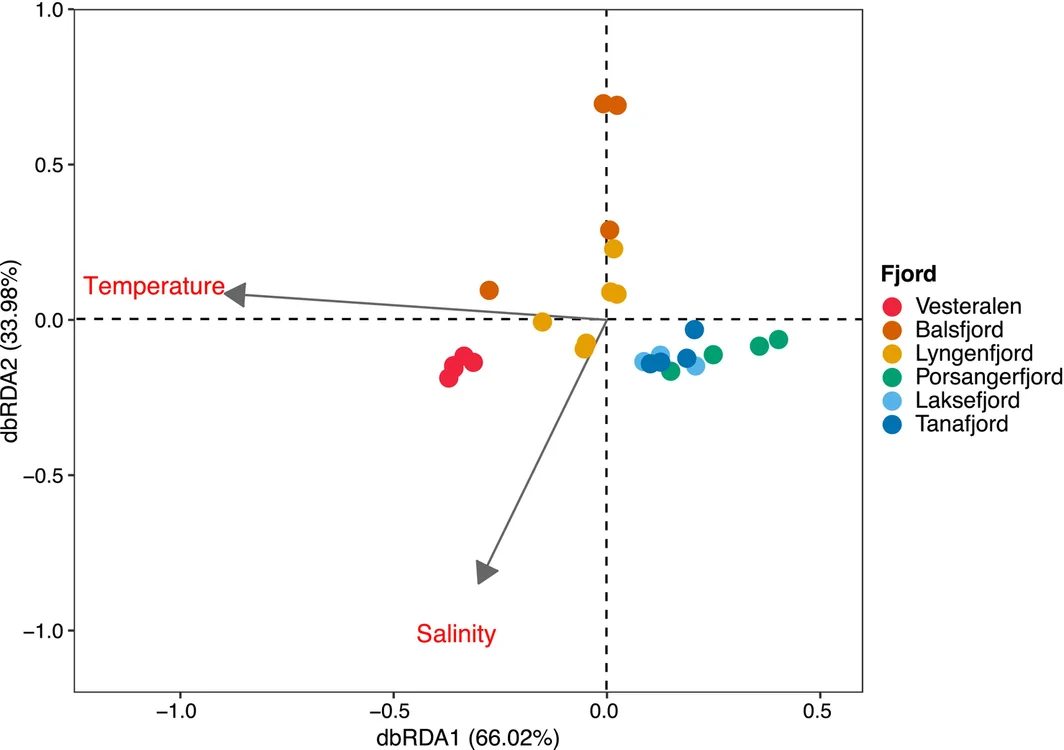
Distance‐based redundancy analysis (dbRDA) model for significant abiotic factors driving differences in the protist community composition in Northern Norway fjords.

A change in the proportion of the different taxonomic divisions was evident along the various fjords. Dinoflagellate relative abundance increased in the fjords from south to north, with high relative abundances at the most northern fjords, Porsangerfjord, Laksefjord, and Tanafjord (avg. ~50% of the total community composition; Figure 5A). Stramenopiles were abundant only in Vesterålen and less common in the northern region (max 20.3% in st28 at outer Tanafjord). Cryptophytes dominated in Vesterålen (max 40%, at st04) and were also abundant in Lyngenfjord, where ASVs assigned to *Plagioselmis prolonga* and *Teleaulax gracilis* were dominant (Figures 5A and 6C). Haptophyta ASVs showed the highest proportions in Balsfjord (55.8% at st10 and 43.6% at st09) and Porsangerfjord (Figure 5A), dominated by those assigned to *C. leadbeateri* and *Phaeocystis pouchetii*, respectively (Figure 5C). Common to all stations was the low contribution of ASVs belonging to the heterotrophic groups Ciliophora, Telonemia, Picozoa, Katablepharidophyta, Centroheliozoa, Choanoflagellida, Fungi, Sagenista, and Opalazoa. Redundancy analysis indicated that temperature and salinity were significant factors (PERMANOVA, *p* < 0.05), explaining changes in community composition at the division level (Figure 5B) and correlating positively with Cryptophyta, Pseudofungi, and Ochrophyta, and negatively with Dinoflagellata and Haptophyta reads. Chlorophyte reads were relatively abundant in the picoplankton fraction, with the highest values in Vesterålen, the inner Lyngenfjord, and Laksefjord. At the same time, they were present in low proportion in the nanoplankton fraction and were very scarce in the microplankton fraction (Figure S6). Haptophyte reads were also present in the picoplankton, mainly in Balsfjord and Porsangerfjord, and in the nanoplankton at st09 and st10 of Balsfjord. Whereas dinoflagellates (including Dinophyceae and Syndiniales) were only relatively abundant in some samples in the picoplankton size fraction, mostly in Tanafjord, their nanoplankton relative abundance increased in the north, attaining around 50% of the total read abundances in the northern fjords. Common in the microplankton samples was a higher contribution from Ochrophyta (photosynthetic heterokonts), Dinophyta, and Ciliophora, with very low or negligible representation from the remaining divisions. Ochrophyta dominated at all Vesterålen stations and at the outer Balsfjord, Lyngenfjord, and Tanafjord. Dinoflagellates were the most abundant in all inner stations except for Lyngenfjord, where ciliates dominated. We found fjord‐specific diversity and differences between the cold Barents Sea fjords and the Norwegian Sea fjords and adjacent coastal area. The intersection matrix indicated that 668 ASVs were present in all studied fjords and divisions (Figure S7). The North‐Eastern fjords shared 47 ASVs, and the South‐Western ones shared 45. The highest number of unique ASVs per fjord was found off Vesterålen (549), followed by Tanafjord, Balsfjord, Lyngenfjord, Porsangerfjord, and Laksefjord (160, 143, 131, 120, 57 ASVs, respectively).

**FIGURE 5:**
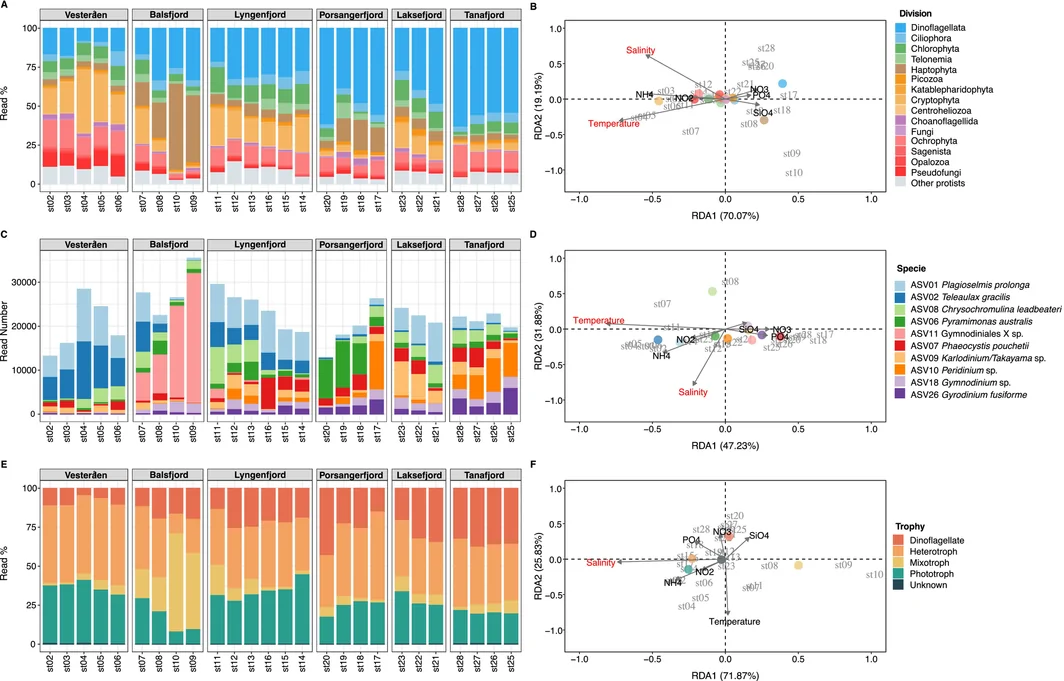
Read abundances among the studied fjords and redundancy analysis of relative abundances in relation to the environmental factors with *p*‐value < 0.05 for taxonomic distribution of protist divisions as proportional abundances (Other protists include: Apicomplexa, Perkinsea, Rhodophyta, Streptophyta, Mesomycetozoa, Cercozoa, Radiolaria, Lobosa, Conosa, Apusomonadidae, Metamonada, and those that could not be classified further than to Alveolata, Archaeplastida, Opisthokonta, Rhizaria and Stramenopila); A and B., Taxonomic distribution of the ten most abundant ASVs (> 30,000 reads per ASV after rarefying); C* and D., and distribution of trophic modes as proportional abundances; E and F. Stations are presented outer inwards the fjords. Black arrows in the RDA plot indicate that the factors are significant (*p* < 0.05) and the red arrows non‐significant (*p* > 0.05) for each given analysis. *Figure 5C is presented in read numbers to provide a direct representation of absolute abundance.

**FIGURE 6:**
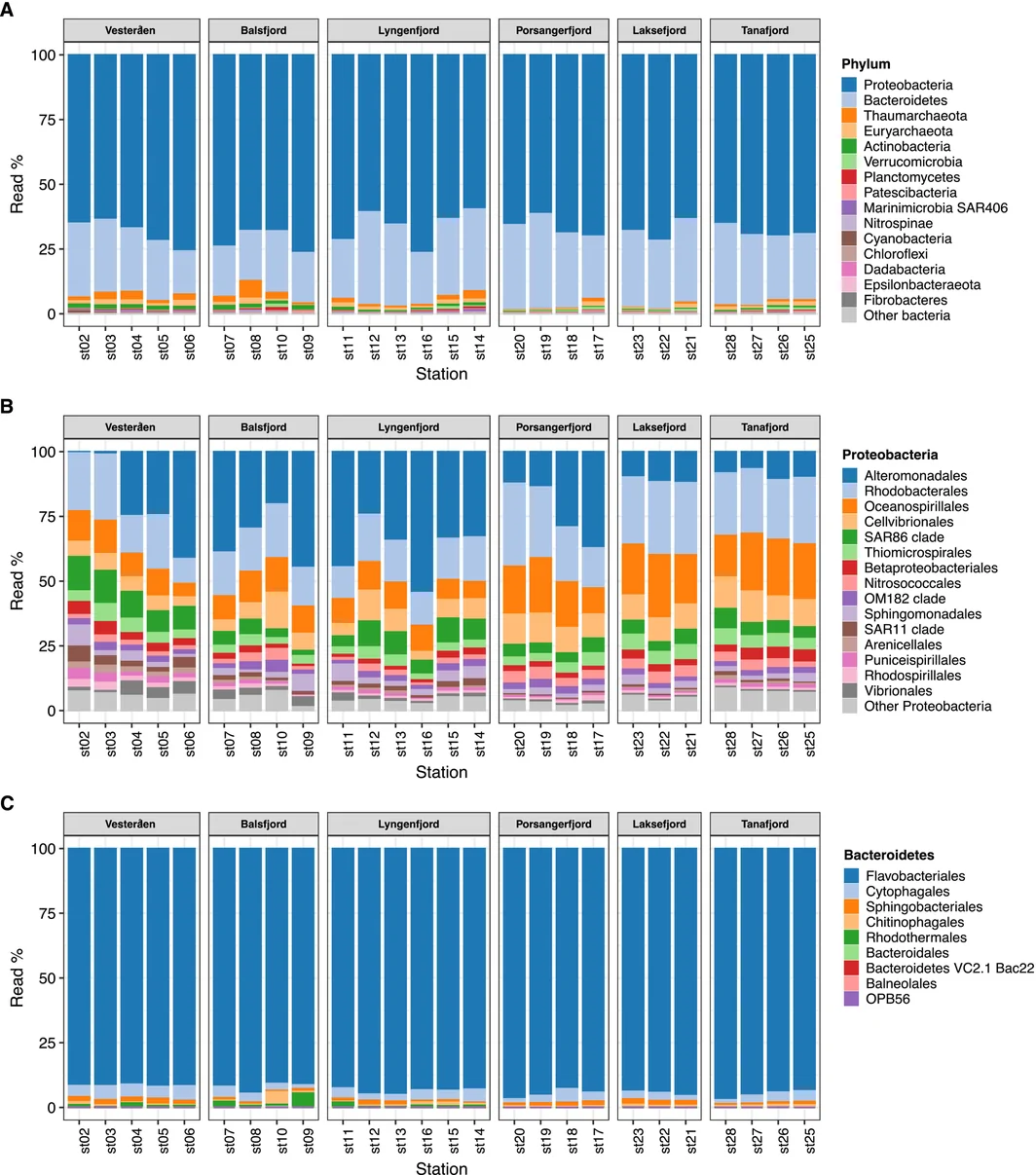
Bacterial read proportional abundances among the studied fjords at (A) Phylum level, (B) Proteobacteria, and (C) Bacteroidetes.

The ten most abundant ASVs were present at all stations studied (Figure 5C). In Figure 5C, the read numbers are presented to provide a direct representation of the absolute abundance of each ASV. The most abundant ASV encompassing all size groups was assigned to the cryptophyte *P. prolonga* (ASV01). *Plagioselmis prolonga* showed similar within‐fjord abundances in Vesterålen, Lyngenfjord, and Laksefjord. In contrast, Balsfjord differed, with higher abundance in the outer fjord and lower in the inner fjord, dominated by the blooming *C. leadbeateri* . The second most abundant ASV, assigned to *T. gracilis* (ASV02), also had the highest abundances in Vesterålen and Lyngenfjord, whereas *C. leadbeateri* (ASV08) dominated in Balsfjord and *P. pouchetii* (ASV07) in Porsangerfjord. Redundancy analysis indicated that the proportions of ASVs were differently affected by the physical–chemical parameters (Figure 5D). *Plagioselmis prolonga* and *T. gracilis* were positively correlated with all three parameters, whereas *C. leadbeateri was* negatively correlated with all three parameters. *Plagioselmis prolonga* and *T. gracilis* were positively correlated with each other and negatively correlated with *P. pouchetti* and *Gyrodinium fusiforme* (Figure S4). Also, *P. prolonga* was positively correlated with *Pyramimonas australis, P. pouchetti*, and *G. fusiforme* .

### 3.4 Trophic Modes Along the North Norway Coast

Looking at the distribution of trophic modes of protists (putative autotrophs, mixotrophs, heterotrophs) along the Northern Norway Coast, we found that heterotrophs dominated all fjords (avg. 35%–50% per fjord) except in Balsfjord (28%, Figure 5E), where mixotrophs dominated. An apparent increase in the proportions of dinoflagellates_UT (dinoflagellates not assigned to autotrophs, mixotrophs, or heterotrophs) in the northernmost fjords was observed, as well as a decrease in the autotrophic protists. Mixotrophs were present, but in low proportions in all fjords except Balsfjord, where the massive bloom of the mixotroph *C. leadbeateri* took place. Mixotrophs seemed to be most abundant in less saline waters (salinity 32–33, Table S1; Figure S1), whereas dinoflagellates_UT were most abundant in colder waters, as indicated by the redundancy analysis (Figure 5F). Relative abundance of autotrophs was negatively correlated with mixotrophs and dinoflagellates_UT, whereas positive correlations were found between heterotrophs and groups with unknown trophic status (Figure S4). A positive correlation was also found between mixotrophs and dinoflagellates_UT, suggesting that most of these dinoflagellates could be mixotrophs.

### 3.5 Prokaryote Diversity by Metabarcoding and Co‐Occurrences With Protists

Co‐occurring prokaryotic diversity was investigated by metabarcoding targeting the 16S rRNA gene. Proteobacteria and Bacteroidetes were the most abundant prokaryotic phyla at all studied stations (avg. 68% and 27% of total abundances, respectively; Figure 6A). The remaining bacterial phyla and archaea accounted for only 1.5%–12.8% of the total reads. Within the Proteobacteria phylum, Alteromonadales, Rhodobacteriales, and Oceanospirales contributed most to the surface communities. Strikingly, Alteromonadales differed in relative abundances of < 1% of Proteobacteria in the westernmost stations in Vesterålen (st2, st3) to > 50% of Proteobacteria in Lyngenfjord (st16). Laksefjorden and Tanafjorden showed the lowest relative abundances of Alteromonadales and the highest relative abundances of Rhodobacteriales among the Proteobacteria. Distribution patterns between fjords became more evident when we examined the most abundant prokaryotic ASVs (Figure S8). Here, several ASVs showed fjord‐specific relative abundance patterns. For example, two ASVs belonging to Rhodobacterales (ASV04b) and Oceanospirillales (ASV10b) showed elevated relative abundances in the three northern fjords, compared to low (< 2%) relative abundances in the three western fjords. In contrast, ASV01b, annotated as Alteromonadales, was least abundant in Laksefjord, Tanafjord, and stations 02 and 03, whereas it showed mean relative abundances of > 15% in other fjords.

Detailed correlation analyses were performed between the ten most abundant protist ASVs and the 20 most abundant Proteobacteria and Bacterioidetes ASVs (Figure 7). Several of the most abundant protist ASVs showed significant correlation with the most abundant prokaryotic ASVs. For example, *P. prolonga* (ASV01b) showed a strong positive correlation with *Marinoscillum* (ASV31b) and with Flavobacteriaceae (ASV10b). *T. gracilis* (ASV02b) also positively correlated with *Marinoscillum* (ASV31b), and *P. pouchetii* (ASV07b) with OM182 clade (ASV33b), Cryomorphaceae (ASV12b), and *Polaribacter* (ASV48b). On the contrary, *T. gracilis* (ASV02b) showed a pronounced negative correlation with, for example, Aurantivirga (ASV19b), *Polibacter* (ASV29b), and *Gyrodinium fusiforme* (ASV26b), as well as both Vibrio (ASV32b) and *Pseudofulvibacter* (ASV29b).

**FIGURE 7:**
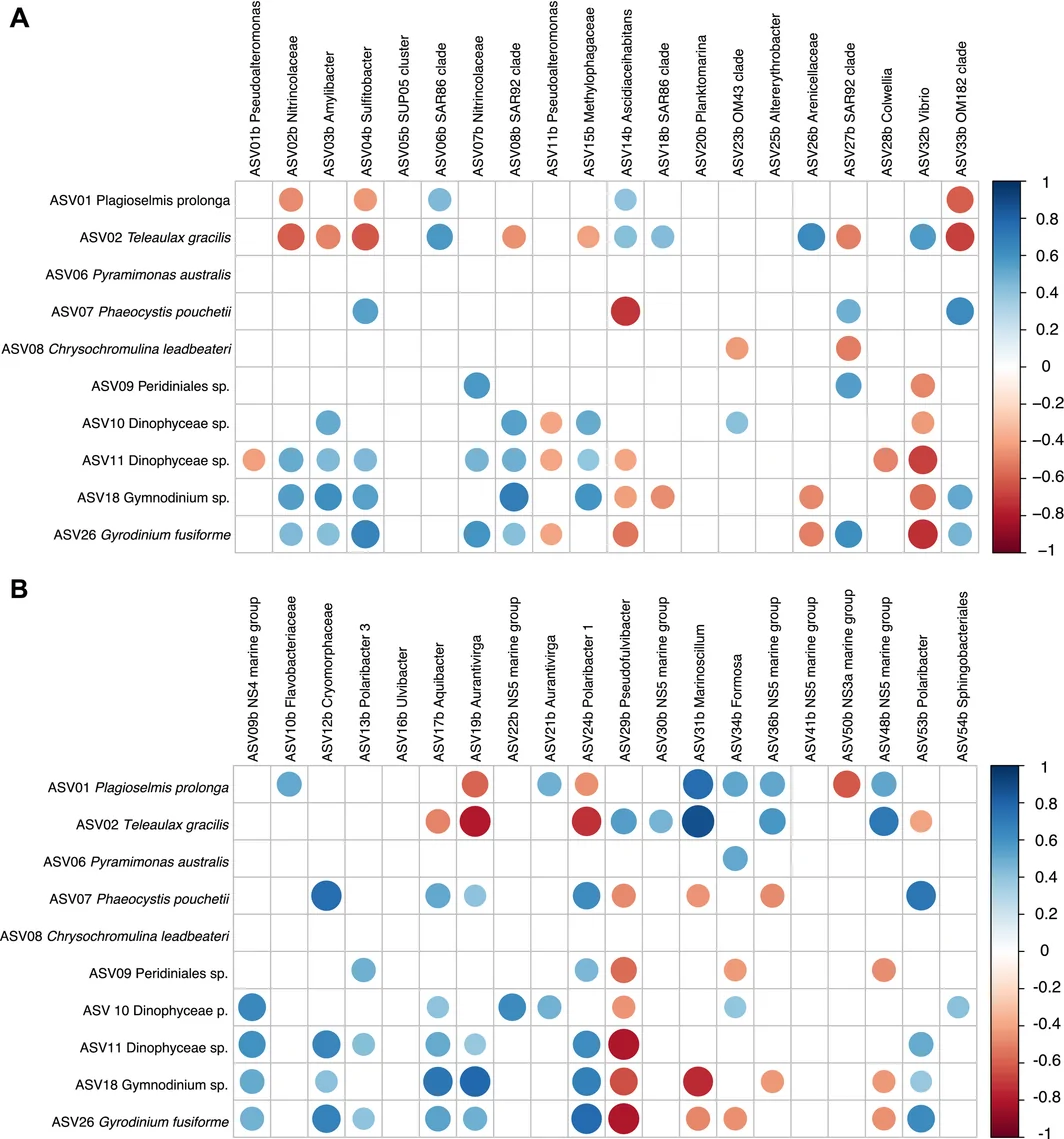
Spearman correlation matrix at *p*‐values < 0.05 between the 10 most abundant eukaryotic ASVs and 20 most abundant Proteobacteria; A. and Bacteroidetes; B. The symbol size is inversely related to the *p*‐value. Blue tones indicate positive correlations, whereas red tones indicate negative ones. “Other Proteobacteria” include Proteobacteria orders with fewer than 30,000 reads each order.

## 4 Discussion

Protist diversity and abundance are fundamental to understanding the structure and function of marine food webs. Here, we present an exhaustive spatial analysis of protists and associated prokaryotic communities along six sub‐Arctic transects on the Scandinavian coast using metabarcoding. Our findings reveal pronounced variation in protist community diversity along the Norwegian Coast.

### 4.1 Protist Community and Major Taxa in the Northern Norwegian Coast

In general, the protist community composition in the Northern Norwegian Coast resembled post‐bloom communities (Degerlund and Eilertsen 2010; Eilertsen et al. 1981), with Alveolates, “Hacrobians” (including Haptista and Cryptista), and Stramenopiles dominating during the sampling season. The analysis indicated that 20.5% of the ASVs were present across all size fractions. Potential overlapping of ASVs and even species across size fractions is common. It can arise from several natural and technical factors: (i) chain formation of protist cells of varying sizes, (ii) aggregates containing cells of different taxa and sizes, (iii) prey signals from food vacuoles of larger cells and fecal pellets, and (iv) but also from cell lysis, broken cells due to filtration forces and filter clogging, all of which can result in the crossover of cells and the introduction of biases in the distribution of ASVs across different size fractions (i.e., Marañón 2015 and others). Additionally, differences in sequencing depth among samples may influence which ASVs are detected: low‐abundance taxa may be lost when sequencing coverage is shallow, and copy‐number variation in rRNA genes can inflate the apparent abundance of some groups, such as for Alveolates. These factors should be kept in mind when interpreting patterns of dominance or differences among size fractions.

The distribution of ASV abundance in protist communities often shows few ASVs with high abundance and many with low abundance (Logares et al. 2014), a pattern consistent with that observed in our study. Our results show dominance of Alveolata in ASV richness and read counts, followed by Stramenopiles and Rhizaria, which is consistent with previous findings in Norwegian coastal ecosystems. For example, studies by Seeleuthner et al. (2018) found that Alveolata and Stramenopiles were the most abundant phyla in Norwegian coastal waters and the Norwegian fjords. Below, we outline the main phyla in our study, the species and ASVs, and their ecological roles in marine ecosystems.

Dinoflagellates (Alveolata/Dinoflagellata) were the most abundant and diverse phylum in the dataset, aligning with observations from another rDNA metabarcoding study of the Norwegian coast (i.e., Gran‐Stadniczeñko et al. 2019). Globally, dinoflagellates encompass probably far more than 2400 species, making them a major component of freshwater and marine microbial food webs (Gómez 2012). Their prominence is particularly notable in estuaries and coastal marine waters, where they often reach peak abundances (Not et al. 2012). However, ribosomal RNA (rRNA) copy numbers in protists are highly variable among protists and within groups such as dinoflagellates and ciliates, exhibiting extremely high copy numbers, potentially over‐inflating their estimated abundance (Ruvindy et al. 2023; Thornhill et al. 2007; Hou et al. 2019; Louca et al. 2018; Gran‐Stadniczeñko et al. 2019). In general, copy number variability can also affect molecular ecological studies employing metabarcoding and quantitative PCR, requiring caution in interpreting data based on rRNA genes (Ruvindy et al. 2023). While rRNA gene copy number variation can complicate the interpretation of abundances between taxa, observations of changes in the relative abundance of ASVs assigned to individual taxa, such as dinoflagellates, between samples remain informative. Our dataset shows that dinoflagellate relative abundances increase from south to north, dominating the protist community in subarctic fjords. The orders Gymnodiniales and Peridiniales were the most abundant dinoflagellate orders, with species of the heterotrophic genera *Gymnodinium* and *Gyrodinium* emerging as the most prevalent genera in the studied areas. Recent findings by Aalto et al. (2022) highlighted the prevalence of *Gymnodinium*‐like dinoflagellates under low‐irradiance and low‐nutrient conditions, such as those under heavy sea ice in the Barents Sea. This observation resonates with our dataset, in which *Gymnodinium* dominated, especially under nutrient‐variable and low‐irradiance conditions of the northern fjords. Moreover, Aalto et al. (2022) reported a strong negative relationship between these taxa and salinity, a pattern also evident in our dataset. Yet, there is still a lack of molecular references in databases, which does not resolve them well at the species level, leaving the most abundant ASVs only assigned to “Gymnodiniales sp.,” possibly due to the challenges in the cultivation (i.e., Andersen 2005; Camacho et al. 2011; Gallardo‐Rodríguez et al. 2016) and identification of these heterotrophic or mixotrophic dinoflagellates, which hinder detailed ecological studies on this important taxon and might be compensated for in the future by single‐cell isolations and associated genetic analyses.

The most abundant ASV was assigned to the cryptophyte *P. prolonga* (Cryptophyta, ASV01), which is the haploid stage of the life cycle of *Teleaulax amphioxeia* (Altenburger et al. 2020). One of the most important ecological aspects of *P. prolonga* is its ability to adapt to changing environmental conditions (Šupraha et al. 2014). *Plagioselmis prolonga* was found to have the highest relative abundances in Vesterålen, Lyngenfjord, and Laksefjord, with relatively similar proportions within fjords, demonstrating its capacity to adapt to a wide range of temperatures, salinities, and light intensities. Although Cryptophyta is less studied in marine ecosystems, their dominance in read numbers in our study is consistent with findings by Hamilton et al. (2021), who identified Cryptophyta as a major component of marine protist communities in the Arctic. Their dominance in our data suggests that Cryptophyta, particularly species such as *P. prolonga* , play a crucial ecological role in the sub‐Arctic marine ecosystems, potentially serving as key primary producers.

The haptophytes *P. pouchetii* and *C. leadbeateri* were also among the most abundant, with *C. leadbeateri* showing exceptionally high densities in st09 and st10 in Balsfjorden, corroborated by the high chl‐a concentration in st10 (17.4 μg L^−1^), compared to the other stations where chl‐a never exceeded 5 μg L^−1^. *Chrysochromulina leadbeateri* is recognized as a common member of the marine nanoplankton in Northern Norway, contributing to phytoplankton blooms and being associated with harmful algal blooms (Aalto et al. 2023; John et al. 2022; Otte et al. 2025). A more comprehensive study of the devastating effects of the *C. leadbeateri* bloom reported in the HE533 study is detailed in John et al. (2022), which stands as the most harmful algal event recorded in the region (Samdal and Edvardsen 2020). Our data show that the exceptionally high densities of *C. leadbeateri* observed in Balsfjorden, particularly at stations st09 and st10, represent a distinct bloom scenario (John et al. 2022; Otte et al. 2025). This bloom event likely led to Balsfjord forming a distinct cluster, separating it from other locations. While members of *Chrysochromulina* are recognized as common members of the marine nanoplankton in all seas, and *C. leadbeateri* appears to have a wide geographical distribution (Fon et al. 2024), fish‐killing blooms of this species have only been recorded from Norway, underscoring the ecological significance and potential harmful impacts of these specific algal bloom events in Norwegian coastal waters.

Further, *P. australis* (ASV 6), a member of the Chlorophyta, was one of the most abundant ASVs in our dataset, present across all studied fjords. Despite its prevalence, the total abundance of Archaeplastida—comprising Chlorophyta, Rhodophyta, and Streptophyta—was notably low, echoing the findings from the Ocean Sampling Day (OSD) study (Tragin and Vaulot 2018). While the OSD study indicates that Chlorophyta represents only 29% of global photosynthetic reads and is mainly prevalent in oligotrophic stations and along the European Atlantic coast, their widespread distribution and dominance in particular localities highlight their potential regional significance. The high abundance of *P. australis* in our observations, despite the generally low total abundance of Archaeplastida, underscores the species' possible key role in regional carbon cycling dynamics. This suggests that, in specific ecosystems, *P. australis* and similar Chlorophyta members could be key primary producers, contributing significantly to local ecological processes and carbon fluxes.

### 4.2 Trophic Modes of the Protist Communities Between Fjords in Temperate/Sub‐Arctic

Ratios of the different trophic modes (autotrophs, mixotrophs, heterotrophs, and dinoflagellates_UT) were similar within each fjord. Notably, heterotrophs dominated in all fjords except for Balsfjord, where the *Chrysochromulina leadbeateri* bloom occurred, leading to a dominance of mixotrophs. This coincides with results from the Skagerrak (Gran‐Stadniczeñko et al. 2019) and the TARA Oceans expedition, based on metabarcoding (De Vargas et al. 2015), which indicate that heterotrophic protists are typically more abundant in marine systems than autotrophic (Hörstmann et al. 2024). The relative abundance of autotrophs was highest in the western fjords compared to the northern ones. In contrast, the opposite pattern was found for dinoflagellates_UT (dinoflagellates Unassigned Trophy), where the temperature was colder. Our redundancy analysis showed a negative correlation between autotrophs and a positive correlation between dinoflagellates‐UT and mixotrophs, suggesting that many of the unassigned dinoflagellates are mixotrophs. In addition, this negative correlation between groups does not necessarily reflect competition alone; it may also arise from predator–prey dynamics or from adaptations to different physical conditions. In our dataset, a marked difference in the proportion of dinoflagellates was found between Vesterålen and the five studied fjords. The lowest proportions occurred at Vesterålen and outer fjord stations, whereas the highest proportions were observed in the inner fjords. This could be because dinoflagellates do not thrive best in turbulent waters but prefer stratified, weakly turbulent conditions (Barton et al. 2015), as strong turbulence can impact their ability to swim toward light or away from prey (Durham et al. 2009). Thus, part of the negative correlation with other groups may reflect differing responses to local hydrodynamics rather than direct biological interactions.

Our findings emphasize the significant yet unresolved role of dinoflagellates, whose exact trophic status remains unclear. Further detailed studies are needed to fully understand their impact on ecosystem balance. Additionally, greater autotrophic dominance in the western fjords than in the northern ones suggests distinct ecosystem responses to environmental changes (e.g., nutrient concentrations), which is essential for forecasting the impacts of climate change. Harmful algal blooms (HABs), such as the *C. leadbeateri* observed in Balsfjorden, disrupt nutrient cycling, trophic dynamics, and primary production by altering nutrient availability, causing toxin accumulation in marine food webs, and creating oxygen‐depleted dead zones (Karlson et al. 2021). Monitoring these variations is essential for understanding how shifts in trophic dynamics influence carbon cycling and overall ecosystem health in these sensitive marine environments. However, it is worth noting that misinterpretations concerning trophic mode assignation, arising from limitations in available databases and the complexity of protist ecology (but see Schneider et al. 2020, and Mitra et al. 2023), could occur due to incomplete information, taxonomic ambiguity, ecological variability, methodological constraints, and the need for cautious interpretation.

### 4.3 Abiotic and Biotic Factors Explaining the Differences in Protist Community Composition Within and Among the Investigated Fjords

Subpolar regions, characterized by harsher conditions, typically have lower protist diversity and abundance than temperate areas (Ibarbalz et al. 2019). This pattern is evident in our findings, where the overall diversity in the western fjords was higher. Sub‐Arctic regions are generally characterized by lower temperatures, reduced light levels, and higher nutrient levels compared to temperate regions (Moore et al. 2013; Reagan et al. 2024). Consequently, subpolar protist communities are often dominated by species well‐adapted to these conditions. Our study's redundancy analysis (RDA) and Permutational Multivariate Analysis of Variance (PERMANOVA) have revealed distinct protist community structures between stations in the western and Barents Sea‐oriented fjords. Sampling depth and rarefaction can influence NMDS ordinations and, therefore, warrant careful consideration in microbial ecology studies. NMDS is relatively robust because it is based on rank‐order dissimilarities rather than absolute abundances and does not assume a specific data distribution. As a result, it is less sensitive to differences in sequencing depth than many alternative methods. However, rarefaction may still affect NMDS results by reducing the effective sampling depth and increasing stochastic loss of low‐abundance taxa. In addition, filtration‐related biases, such as the underrepresentation of larger protists due to filter clogging or cell lysis during sampling, may further influence community composition independently of the ordination method (Weiss et al. 2017; McMurdie and Holmes 2014). However, rarefaction remains beneficial in this context as it normalizes sequencing depth across samples, reducing the risk of inflated distance/similarity measures driven by unequal sampling effort rather than true biological variation. This normalization supports more reliable ecological interpretation by minimizing the influence of technical bias on ordination patterns.

Temperature and salinity emerged as significant factors influencing northern Norway's protist communities, accounting for 28% of the total observed variation. Oceanographic boundaries, shaped by temperature and salinity gradients and by varied water masses, are instrumental in distinct habitats in the marine environment, influencing microbial diversity within the marine ecosystems of the northern Atlantic and the Barents Sea. For instance, the colder water masses of the Barents Sea may limit microbial diversity and, here, in combination with the nutrient regime, the formation of high biomass, as seen in similar cold marine environments (Aalto et al. 2022). In contrast, the warmer waters of Hudson Bay are likely to support a more diverse microbial community (Jacquemot et al. 2021). Furthermore, marine plankton exhibit remarkable adaptability to environmental changes, which could lead to ecosystem shifts in response to evolving conditions (Ji et al. 2010; Chivers et al. 2017). This adaptive capacity is central to the resilience and diversity of microorganisms across the spatial and temporal spectrum of the northern Atlantic and Barents Sea regions. Thus, our findings align well with the notion that temperature is a primary driver in marine ecosystems.

Nutrient limitations and changes in the ratios of key nutrients such as nitrogen, phosphorus, and silica affect community composition and species abundance, including trophic interactions (Jacquemot et al. 2021; Bazin et al. 2014). This could increase the abundance of protists that feed on phytoplankton, such as ciliates and dinoflagellates. On the other hand, low nutrient levels can limit phytoplankton growth and reduce the abundance of protists that depend on them for food. Additionally, different types of protists may have distinct nutrient requirements, leading to the dominance of certain types of protists in areas with specific nutrient levels. PO_4_^−^, SiO_4_, NO_2,_ NO_3,_ and NH_4_ exhibited a spatial variability along the studied stations, with potential implications for primary production and eutrophication. In our RDA analysis, the five studied nutrients collectively explained about 17% of the variation in the protist community composition. However, their individual contributions are not statistically significant, except for NO_3_, which shows marginal significance (*p*‐value = 0.085). This suggests that although nutrients play a role, other factors are likely more influential in shaping the community structure. This observation aligns with the broader understanding of nutrient dynamics in marine ecosystems, where variations in nutrient levels can impact protist communities' abundance and composition (Jacquemot et al. 2021). Most of the nutrients in our dataset show positive correlations with one another, suggesting that disentangling the effects of individual nutrients is challenging and likely also reflects co‐limitation (e.g., Bannon et al. 2022). Yet, in the db‐RDA analysis, the order in which the environmental parameters are entered can influence the results. Specifically, the significant influence of temperature and salinity in the analysis highlights their dominant role in shaping the protist community structure, potentially overshadowing the collective influence of the overall nutrient load.

Although this study focused on protists, we also observed a consistent pattern in the associated bacterial community between fjords, with some distinct bacterial ASVs found in specific fjords only. However, at lower taxonomic ranks, all stations included high relative abundances of *Alteromonadales*, *Rhodobacterales*, and *Bacteroidetes*. Bacteria in these groups are typically observed during spring in temperate and arctic areas and during phytoplankton blooms (Aalto et al. 2023; Buchan et al. 2014; Wietz et al. 2021). They play key roles in the microbial loop and organic matter recycling and are themselves shaped by biotic interactions, substrate succession, and other environmental drivers (Buchan et al. 2014; Bunse and Pinhassi 2017; Needham and Fuhrman 2016; Teeling et al. 2016).

Taken together, the relative abundances of different protist groups in each studied fjord result from a complex interplay between various biotic and abiotic environmental factors. Understanding these factors can help explain differences in protist community composition. We identified that temperature, salinity, and phosphorus were major drivers of protist community structure in spring/summer. Patterns of co‐occurrence with other species were also observed, but these cannot be interpreted as causal interactions within the scope of this study.

## 5 Conclusions

In conclusion, the study of protist community composition in subpolar and temperate regions during early summer revealed distinct differences in diversity and composition driven by region‐specific environmental conditions and current systems. These findings highlight the complexity of biological and environmental interactions at local scales. The polar fjords in Finnmark County were characterized by lower temperatures, light levels, and lower nutrient availability, conditions that may be associated with the observed dominance of heterotrophic flagellates and ciliates. In contrast, the western coastal area and fjords had higher temperatures and nutrient availability, resulting in a higher relative abundance of phototrophic phytoplankton, including diatoms, haptophytes, dinoflagellates, and cryptophytes. The study of the Northern Norwegian fjords and coastal ecosystems also highlighted the dominance of dinoflagellates and the unique bloom of the mixotrophic haptophyte nanoflagellate *C. leadbeateri* in Balsfjorden. Our results highlight the importance of accounting for local environmental conditions that shape protist community composition to fully understand the ecological dynamics of these systems.

## Supporting information


**Table S1:** Environmental data for all sampled stations at HE533 Cruise
**Table S2:** Nutrient values of the HE533 cruise at (A) 3 m, (B) DCM (3‐28 m), and (C) 40 m.
**Table S3:** Summary of the HE533 cruise ASVs and number of Reads before and after rarefaction.
**Table S4:** Summary of Distance‐Based Redundancy Analysis (dbRDA) results for the significant environmental parameters in the HE533 Study. Df: degrees of freedom, SS: sums of squares, F: Fstatistic.
**Figure S1:** ODV transect plots from CTD data for the studied fjords.
**Figure S2:** (A) DCM NH4 concentration. (B) DCM NO2 concentration. (C) DCM NO3 concentration. (D) DCM PO4 concentration. (E) DCM SiO concentration.
**Figure S3:** Chlorophyll‐a distribution at each station and the 3 sampled depths.
**Figure S4:** Spearman correlation matrix at *p*‐values < 0.05 between the most abundant ASVs, physicochemical parameters at the He533 cruise. The symbol size is inversely related to the pvalue. Blue tones indicate positive correlations whereas red tones indicate negative ones.
**Figure S5:** Treemaps displaying the Northern Norway fjords protist HTS community's taxonomic composition at supergroup and division levels: ASVs richness (above) and proportional read abundance (below). proportional read abundances) among the studied fjords by size fractions. Other protists include: Apicomplexa, Perkinsea, Rhodophyta, Streptophyta, Mesomycetozoa, Cercozoa, Radiolaria, Lobosa, Conosa, Apusomonadidae, Metamonada, and those that could not be classify further than to Alveolata, Archaeplastida, Opisthokonta, Rhizaria and Stramenopila. Stations are presented outer inwards the fjords.
**Figure S7:** Distribution of protist groups ASVs across the five fjords and the VesterÃ¥len area. Total number of Protist ASVs at each fjord or area is shown to the left (gray bars). The vertical bars indicate the number of ASVs unique to a particular fjord (single dark circles) or shared among fjords (dark circles connected by solid lines), as well as the taxa representation by color codes.
**Figure S8:** Taxonomic distribution of major taxonomic prokaryotic ASVs (as proportional read abundances) among the studied fjords by size fractions.

## Data Availability

The data that support the findings of this study are openly available in European Nucleotide Archive (ENA) at https://www.ebi.ac.uk/ena/browser/view/ERP134612, reference number ERP134612.
