# Supplementary material for: Spatial Diversity of Marine Protists Along the Northern Norway Fjords During the Sub‐Arctic Summer
Source: J Eukaryot Microbiol. 2026 Oct 10;73(6):e70133. doi: 10.1111/jeu.70133 (PMC13653249; doi:10.1111/jeu.70133)
Supplement: Supplementary file 1 [file JEU-73-e70133-s001.pdf]

Spatial diversity of marine protists along the Northern Norway Fjords during the sub-Arctic summer

SUPPORTING INFORMATION

Table S1. Environmental data for all sampled stations at HE533 Cruise

Sample	Fjord	Date	Latitude	Longitude	Pooled Depth [m]	DCM [m]	Temperature [°C]	Salinity [PSU]	PO_4 [µM]	SiO [µM]	NO_2 [µM]	NO_3 [µM]	NH_4 [µM]
st02	Vestrålen	23.05.2019	68.83	14.3071	3 - 40	15	6.55	34.1	0.44	2.97	0.28	5.56	0.80
st03	Vestrålen	23.05.2019	68.4797	14.9552	3 - 40	25	6.13	33.83	0.22	1.36	0.08	1.49	0.66
st04	Vestrålen	24.05.2019	68.6792	15.433	3 - 40	12	5.45	33.88	0.27	1.87	0.09	1.90	1.17
st05	Vestrålen	24.05.2019	68.9446	15.3878	3 - 40	15	6.05	33.94	0.12	1.58	0.03	0.52	0.34
st06	Vestrålen	24.05.2019	69.1665	15.4146	3 - 40	15	6.36	34.24	0.29	1.63	0.15	3.35	0.70
st07	Balsfjord	25.05.2019	69.6376	17.7832	3 - 40	8	5.84	34.04	0.33	2.13	0.15	3.71	0.88
st08	Balsfjord	25.05.2019	69.5092	18.3193	3 - 40	12	5.49	33.64	0.45	2.99	0.14	6.56	0.45
st09	Balsfjord	26.05.2019	69.2602	19.3616	3 - 40	8	5	32.05	0.38	3.82	0.13	5.02	0.52
st10	Balsfjord	26.05.2019	69.428	19.0239	3 - 40	12	5.28	32.7	0.13	3.32	0.03	0.50	0.20
st11	Lyngenfjord	27.05.2019	70.2858	19.4151	3 - 40	15	5.71	34.28	0.36	3.08	0.11	4.25	0.31
st12	Lyngenfjord	27.05.2019	70.0965	19.6279	3 - 40	18	5.72	33.95	0.38	2.97	0.16	4.86	0.91
st13	Lyngenfjord	27.05.2019	70.0748	20.1181	3 - 40	18	5.89	34.11	0.31	2.13	0.14	3.45	0.37
st14	Lyngenfjord	28.05.2019	69.3617	20.0709	3 - 40	13	5.33	32.86	0.29	1.78	0.07	1.67	0.55
st15	Lyngenfjord	28.05.2019	69.5947	20.346	3 - 40	21	4.94	33.94	0.40	1.95	0.15	4.10	0.51
st16	Lyngenfjord	28.05.2019	69.8249	20.462	3 - 40	26	4.87	33.4	0.54	4.36	0.07	6.93	0.33
st17	Porsangerfjord	30.05.2019	70.2668	25.1665	3 - 40	17	2.85	33.41	0.30	4.44	0.07	3.67	0.12
st18	Porsangerfjord	30.05.2019	70.5125	25.4622	3 - 40	28	3.55	33.69	0.49	2.18	0.08	3.53	0.56
st19	Porsangerfjord	31.05.2019	70.7384	25.9076	3 - 40	28	4.41	33.61	0.50	2.78	0.09	6.20	0.55
st20	Porsangerfjord	31.05.2019	70.9216	26.2514	3 - 40	18	4.72	33.99	0.38	1.98	0.12	4.68	0.61
st21	Laksefjord	01.06.2019	70.4729	26.6765	3 - 40	10	3.16	33.97	0.26	7.22	0.07	2.94	0.25
st22	Laksefjord	01.06.2019	70.6751	26.7865	3 - 40	20	4.1	33.76	0.21	11.94	0.07	2.32	0.82
st23	Laksefjord	02.06.2019	70.8592	27.2751	3 - 40	26	4.09	33.76	0.38	1.97	0.12	4.46	0.90
st25	Tanafjord	03.06.2019	70.5119	28.0784	3 - 40	15	4.31	33.04	0.17	1.38	0.06	1.88	0.48
st26	Tanafjord	03.06.2019	70.7005	28.4502	3 - 40	8	4.32	33.81	0.52	2.49	0.17	7.19	0.62
st27	Tanafjord	04.06.2019	70.8681	28.7054	3 - 40	19	4.68	33.72	0.28	2.47	0.05	3.48	0.37
st28	Tanafjord	04.06.2019	71.0284	28.8202	3 - 40	25	5.32	34.23	0.24	1.05	0.07	2.49	0.57

Table S2. Nutrient values of the HE533 cruise at **A**) 3m, **B**) DCM (3-28m), **C**) 40m.

A)

Station ID	DateTime [UTC]	Lat	Lon	Depth [m]	Chl a [µg/L]	PO_4 [µmol/L]	SiO [µmol/L]	NO_2 [µmol/L]	NO_3 [µmol/L]	NH4 [µmol/L]
st01	22.05.2019 06:00	68.484508	14.307497	3	1.076	0.22	0.90	0.10	1.78	0.90
st02	23.05.2019 06:00	68.482738	14.309437	3	1.328	0.14	1.59	0.03	0.62	0.26
st03	23.05.2019 10:48	68.480521	14.955078	3	1.176	0.25	1.23	0.11	1.83	0.93
st04	24.05.2019 05:55	68.678526	15.434114	3	0.618	0.32	1.28	0.16	2.91	0.56
st05	24.05.2019 10:57	68.943952	15.38773	3	2.185	0.27	1.89	0.14	2.44	1.54
st06	24.05.2019 15:58	69.165412	15.416432	3	0.598	0.41	2.38	0.21	5.75	0.92
st07	25.05.2019 06:00	69.635904	17.788022	3	3.040	0.52	2.91	0.27	7.40	0.87
st08	25.05.2019 10:45	69.508013	18.323074	3	2.317	0.16	3.28	0.04	0.89	0.25
st09	26.05.2019 06:00	69.260078	19.360952	4	4.760	0.34	3.04	0.12	4.09	0.35
st10	26.05.2019 11:46	69.427703	19.024301	3	4.127	0.34	1.53	0.11	1.96	1.12
st11	27.05.2019 05:55	70.285977	19.413279	3	2.944	0.49	3.61	0.03	7.69	0.02
st12	27.05.2019 11:00	70.097911	19.629387	3	1.924	0.07	4.19	0.01	0.16	0.03
st13	27.05.2019 15:57	70.075057	20.122844	3	1.079	0.31	1.99	0.14	3.35	0.25
st14	28.05.2019 06:02	69.360503	20.067697	3	0.831	0.58	3.05	0.20	7.85	0.52
st15	28.05.2019 10:53	69.59611	20.347918	3	0.576	0.58	2.81	0.22	7.14	0.83
st16	28.05.2019 15:55	69.825085	20.462746	3	0.487	0.10	1.41	0.00	0.25	0.10
st17	30.05.2019 05:58	70.265298	25.166565	3	2.036	0.38	2.13	0.10	3.78	0.30
st18	30.05.2019 10:57	70.513398	25.461248	3	0.365	0.53	2.75	0.13	6.96	0.60
st19	30.05.2019 15:45	70.739326	25.910249	3	0.440	0.61	4.30	0.05	9.85	0.02
st20	31.05.2019 10:55	70.921648	26.255838	3	0.855	0.08	1.23	0.01	0.21	0.42
st21	01.06.2019 05:59	70.473238	26.676561	3	0.673	0.11	0.77	0.02	0.32	0.21
st22	01.06.2019 10:56	70.674978	26.785278	3	0.818	0.38	1.26	0.11	4.05	1.99
st23	02.06.2019 06:00	70.859123	27.276405	3	0.815	0.61	3.32	0.20	8.46	0.56
st25	03.06.2019 05:55	70.511623	28.07974	3	-	0.06	0.84	0.01	0.33	0.62
st26	03.06.2019 16:29	70.69982	28.450917	3	-	0.10	1.57	0.02	0.31	1.00
st27	04.06.2019 06:00	70.868734	28.702797	3	0.948	0.18	0.75	0.05	1.16	0.36
st28	04.06.2019 09:52	71.028455	28.82046	3	3.767	0.30	0.81	0.09	2.49	1.56

B)

Station ID	DateTime [UTC]	Lat	Lon	Depth [m]	Chl a [µg/L]	PO_4 [µmol/L]	SiO [µmol/L]	NO_2 [µmol/L]	NO_3 [µmol/L]	NH4 [µmol/L]
st01	22.05.2019 06:00	68.484508	14.307497	15	1.401	0.23	0.94	0.10	1.74	0.84
st02	23.05.2019 06:00	68.482738	14.309437	15	1.057	0.62	4.15	0.43	8.69	0.65
st03	23.05.2019 10:48	68.480521	14.955078	25	1.550	0.14	1.21	0.02	0.26	0.19
st04	24.05.2019 05:55	68.678526	15.434114	12	2.207	0.14	1.16	0.01	0.26	0.43
st05	24.05.2019 10:57	68.943952	15.38773	15	4.517	0.10	1.14	0.01	0.12	0.08
st06	24.05.2019 15:58	69.165412	15.416432	15	0.680	0.26	1.42	0.13	2.70	0.69
st07	25.05.2019 06:00	69.635904	17.788022	8	2.916	0.35	2.13	0.18	4.34	1.15
st08	25.05.2019 10:45	69.508013	18.323074	12	1.709	0.52	3.48	0.14	8.56	0.09
st09	26.05.2019 06:00	69.260078	19.360952	12	3.250	0.25	3.32	0.09	3.00	0.23
st10	26.05.2019 11:46	69.427703	19.024301	12	17.427	0.12	1.55	0.01	0.38	0.05
st11	27.05.2019 05:55	70.285977	19.413279	15	2.710	0.44	2.87	0.18	5.53	0.60
st12	27.05.2019 11:00	70.097911	19.629387	18	3.334	0.49	2.88	0.22	6.81	1.26
st13	27.05.2019 15:57	70.075057	20.122844	18	3.864	0.30	2.16	0.14	3.14	0.26
st14	28.05.2019 06:02	69.360503	20.067697	13	1.094	0.21	1.67	0.07	1.11	0.77
st15	28.05.2019 10:53	69.59611	20.347918	21	1.290	0.38	1.78	0.15	3.76	0.58
st16	28.05.2019 15:55	69.825085	20.462746	26	1.472	0.75	6.05	0.10	9.98	0.50
st17	30.05.2019 05:58	70.265298	25.166565	17	2.258	0.06	5.48	0.02	0.19	0.12
st18	30.05.2019 10:57	70.513398	25.461248	28	-	0.60	1.72	0.08	2.29	0.66
st19	30.05.2019 15:45	70.739326	25.910249	28	0.430	0.48	2.26	0.12	5.27	1.00
st20	31.05.2019 10:55	70.921648	26.255838	18	0.715	0.47	2.21	0.15	5.95	0.65
st21	01.06.2019 05:59	70.473238	26.676561	10	0.625	0.09	2.40	0.01	0.42	0.20
st22	01.06.2019 10:56	70.674978	26.785278	20	1.386	0.22	22.23	0.08	2.70	0.69
st23	02.06.2019 06:00	70.859123	27.276405	26	1.314	0.31	1.44	0.09	2.99	1.18
st25	03.06.2019 05:55	70.511623	28.07974	15	0.891	0.08	0.80	0.02	0.37	0.11
st26	03.06.2019 16:29	70.69982	28.450917	8	0.566	0.60	3.42	0.15	8.68	0.27
st27	04.06.2019 06:00	70.868734	28.702797	19	0.961	0.07	1.37	0.01	0.20	0.56
st28	04.06.2019 09:52	71.028455	28.82046	25	4.135	0.08	0.29	0.01	0.20	0.14

C)

Station ID	DateTime [UTC]	Lat	Lon	Depth [m]	Chl a [µg/L]	PO_4 [µmol/L]	SiO [µmol/L]	NO_2 [µmol/L]	NO_3 [µmol/L]	NH4 [µmol/L]
st01	22.05.2019 06:00	68.484508	14.307497	40	0.508	0.16	1.56	0.05	1.12	0.66
st02	23.05.2019 06:00	68.482738	14.309437	40	2.650	0.31	1.90	0.19	3.31	1.28
st03	23.05.2019 10:48	68.480521	14.955078	40	0.158	0.35	1.94	0.20	4.04	1.43
st04	24.05.2019 05:55	68.678526	15.434114	30	0.276	0.44	3.23	0.18	3.84	2.59
st05	24.05.2019 10:57	68.943952	15.38773	40	0.221	0.07	2.08	0.00	0.20	0.14
st06	24.05.2019 15:58	69.165412	15.416432	30	0.478	0.25	1.40	0.13	2.61	0.54
st07	25.05.2019 06:00	69.635904	17.788022	40	0.556	0.27	2.01	0.10	2.40	0.56
st08	25.05.2019 10:45	69.508013	18.323074	40	0.133	0.46	2.24	0.17	5.73	0.99
st09	26.05.2019 06:00	69.260078	19.360952	40	0.220	0.56	4.69	0.19	7.88	0.95
st10	26.05.2019 11:46	69.427703	19.024301	40	0.194	0.08	6.24	0.03	0.19	0.09
st11	27.05.2019 05:55	70.285977	19.413279	40	0.790	0.17	3.13	0.03	0.70	0.02
st12	27.05.2019 11:00	70.097911	19.629387	40	0.981	0.40	2.28	0.17	4.79	0.93
st13	27.05.2019 15:57	70.075057	20.122844	40	1.151	0.33	2.17	0.15	4.05	0.65
st14	28.05.2019 06:02	69.360503	20.067697	40	0.158	0.28	1.46	0.01	0.16	0.25
st15	28.05.2019 10:53	69.59611	20.347918	40	0.411	0.26	1.47	0.10	1.88	0.08
st16	28.05.2019 15:55	69.825085	20.462746	40	0.349	0.71	4.75	0.10	9.82	0.24
st17	30.05.2019 05:58	70.265298	25.166565	40	0.287	0.64	4.16	0.14	9.21	0.01
st18	30.05.2019 10:57	70.513398	25.461248	40	0.394	0.05	2.39	0.00	0.23	0.18
st19	30.05.2019 15:45	70.739326	25.910249	40	0.360	0.31	1.24	0.06	1.46	0.29
st20	31.05.2019 10:55	70.921648	26.255838	40	0.236	0.42	2.09	0.15	5.60	0.67
st21	01.06.2019 05:59	70.473238	26.676561	40	0.082	0.50	14.66	0.16	6.67	0.31
st22	01.06.2019 10:56	70.674978	26.785278	40	0.282	0.05	1.98	0.00	0.16	0.07
st23	02.06.2019 06:00	70.859123	27.276405	40	0.458	0.18	1.14	0.06	1.79	0.74
st25	03.06.2019 05:55	70.511623	28.07974	40	0.562	0.37	2.51	0.15	4.85	0.96
st26	03.06.2019 16:29	70.69982	28.450917	40	0.177	0.50	1.56	0.22	6.55	0.97
st27	04.06.2019 06:00	70.868734	28.702797	40	0.331	0.72	5.73	0.11	11.04	0.06
st28	04.06.2019 09:52	71.028455	28.82046	40	0.762	0.53	3.26	0.20	8.12	0.16

Table S3. Summary of the HE533 cruise ASVs and number of Reads before and after rarefaction:

	ASVs	Reads	ASVs subs	Reads subs
Dada2 output	5,150	13,907,302	-	-
Removed taxa*	919	4,359,324		
Total Protists	4,231	9,547,978	3,441	1,633,762
Picofraction	2,192	1,535,588	1,614	181,324
Nanofraction	3,510	7,083,456	2,702	1,273,220
Microfraction	1947	928,934	1,466	179,218

* Metazoa, Higher Fungi (Ascomycota and Basidiomycota), Embryophyceae, Cryptophyta nucleomorphs and Bacterial SSUs.

Table S4. Summary of Distance-Based Redundancy Analysis (dbRDA) results for the significant environmental parameters in the HE533 Study. *Df*: degrees of freedom, *SS*: sums of squares, *F*: F-statistic.

	Df	SS	F	P-value
Model	2	0.88	4.46	0.001 ***
dbRDA1	1	0.58	5.89	0.001 ***
dbRDA2	1	0.30	3.03	0.002 **
Residual	23	2.26		
Temperature	1	0.55	5.57	0.001 ***
Salinity	1	0.30	3.06	0.004 ***
Residual	23	2.26		

P-values obtained by 999 permutations. * Significant P-values.

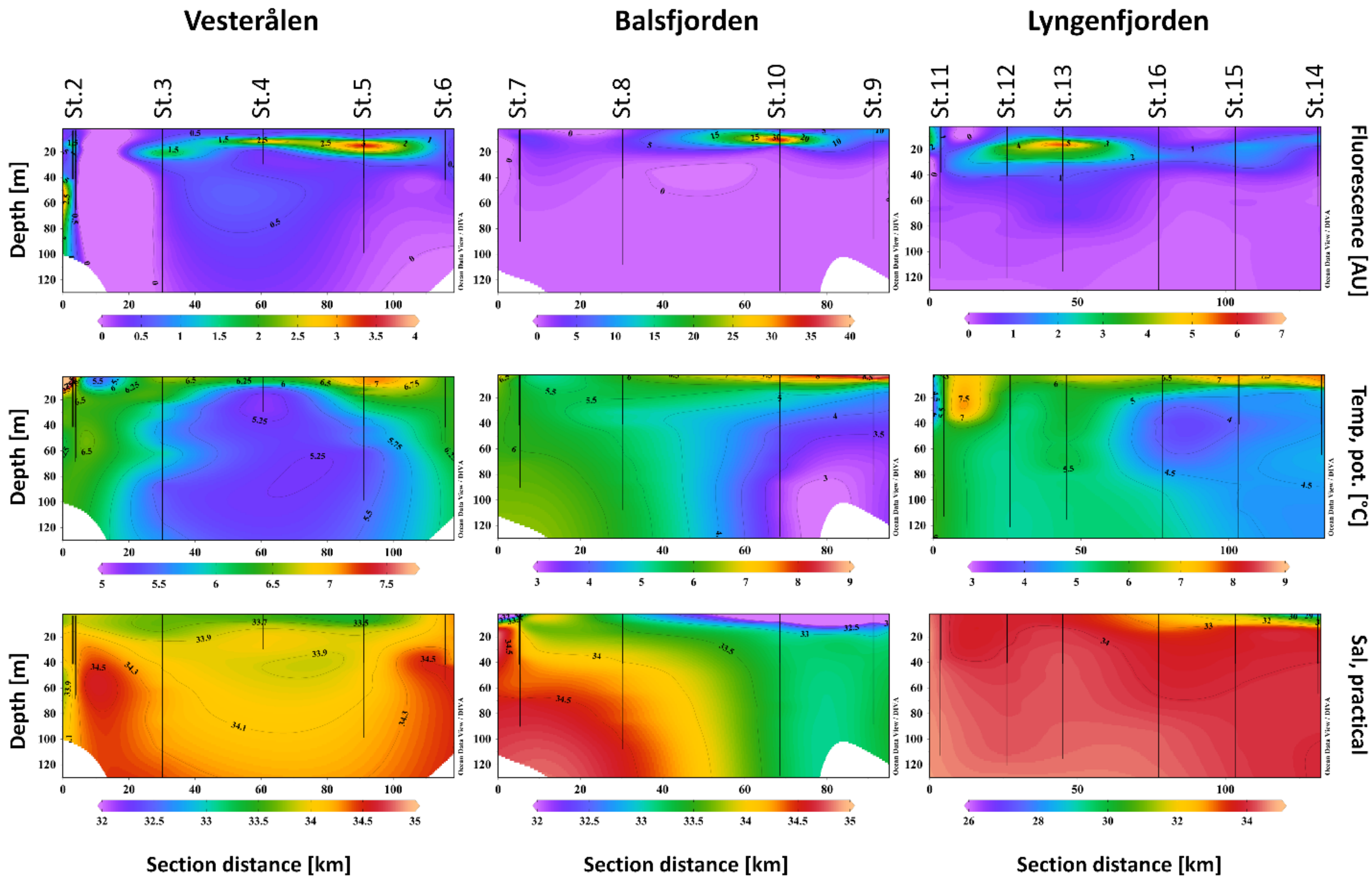

Vesterålen
Balsfjorden
Lyngenfjorden
St.2
St.3
St.4
St.5
St.6
St.7
St.8
St.10
St.9
St.11
St.12
St.13
St.16
St.15
St.14
Depth [m]
Fluorescence [AU]
Temp, pot. [°C]
Sal, practical
Section distance [km]

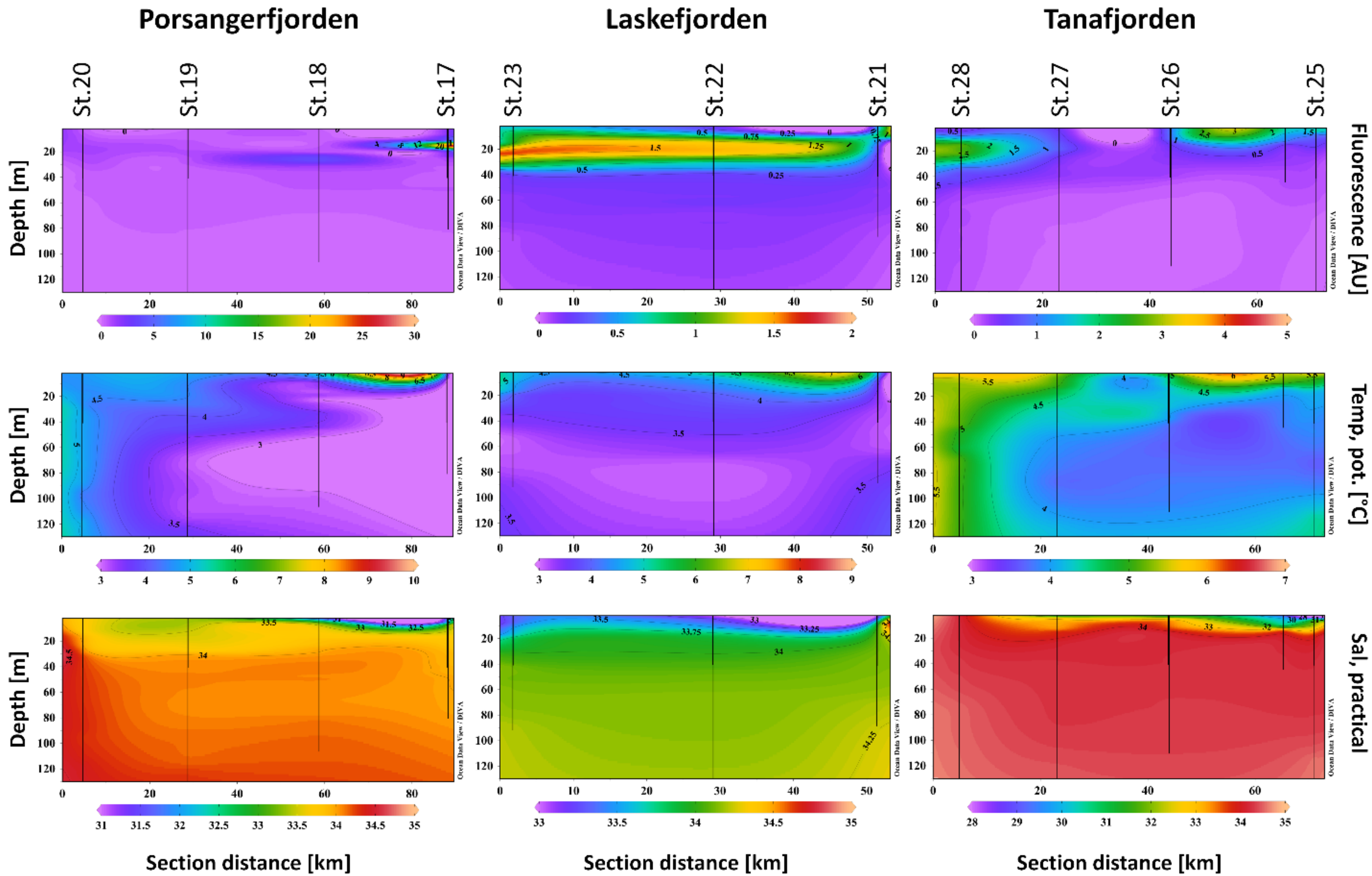

Figure S1. ODV transect plots from CTD data for the studied fjords.

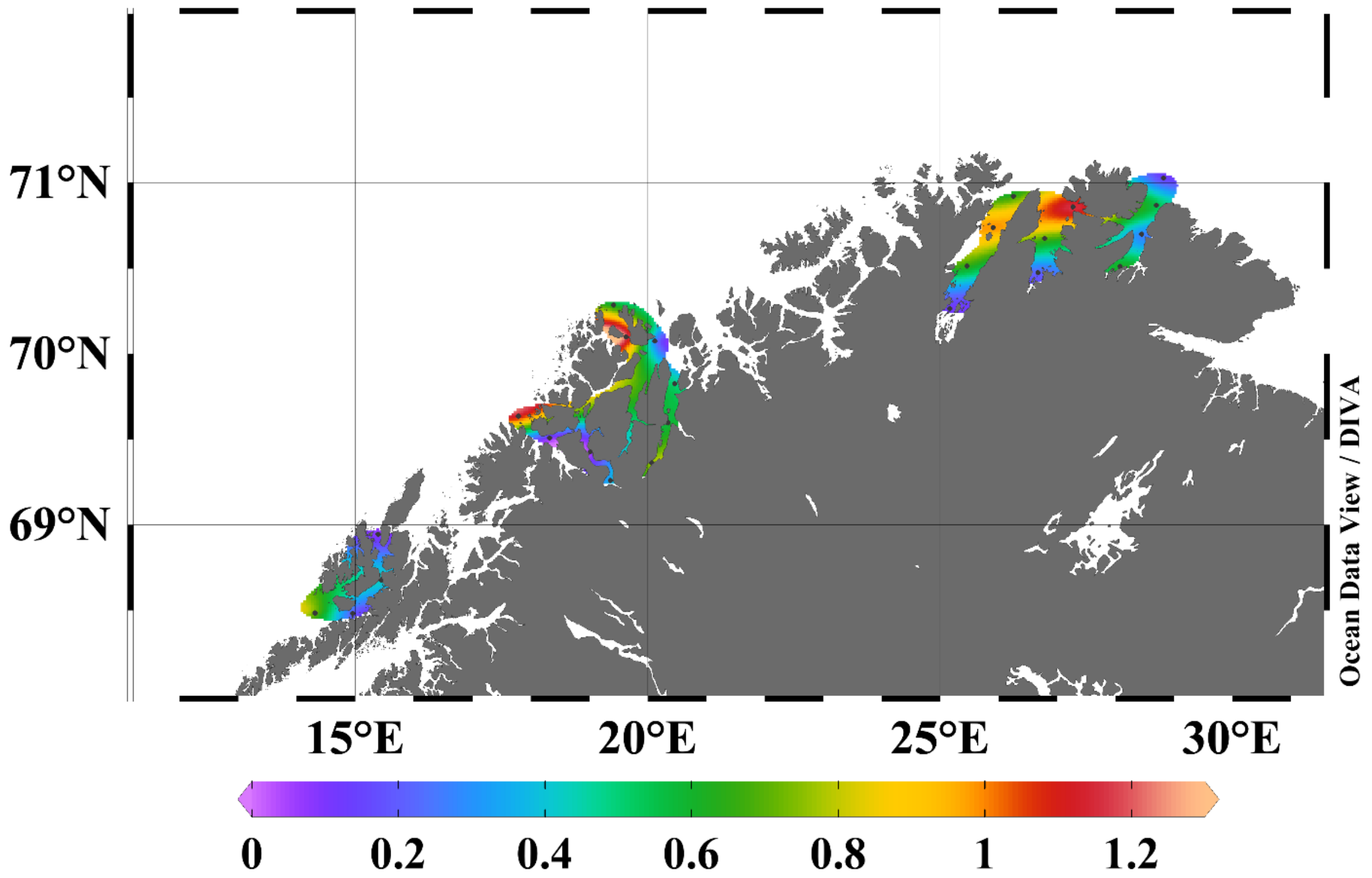

Figure S2. A) DCM NH_4 concentration

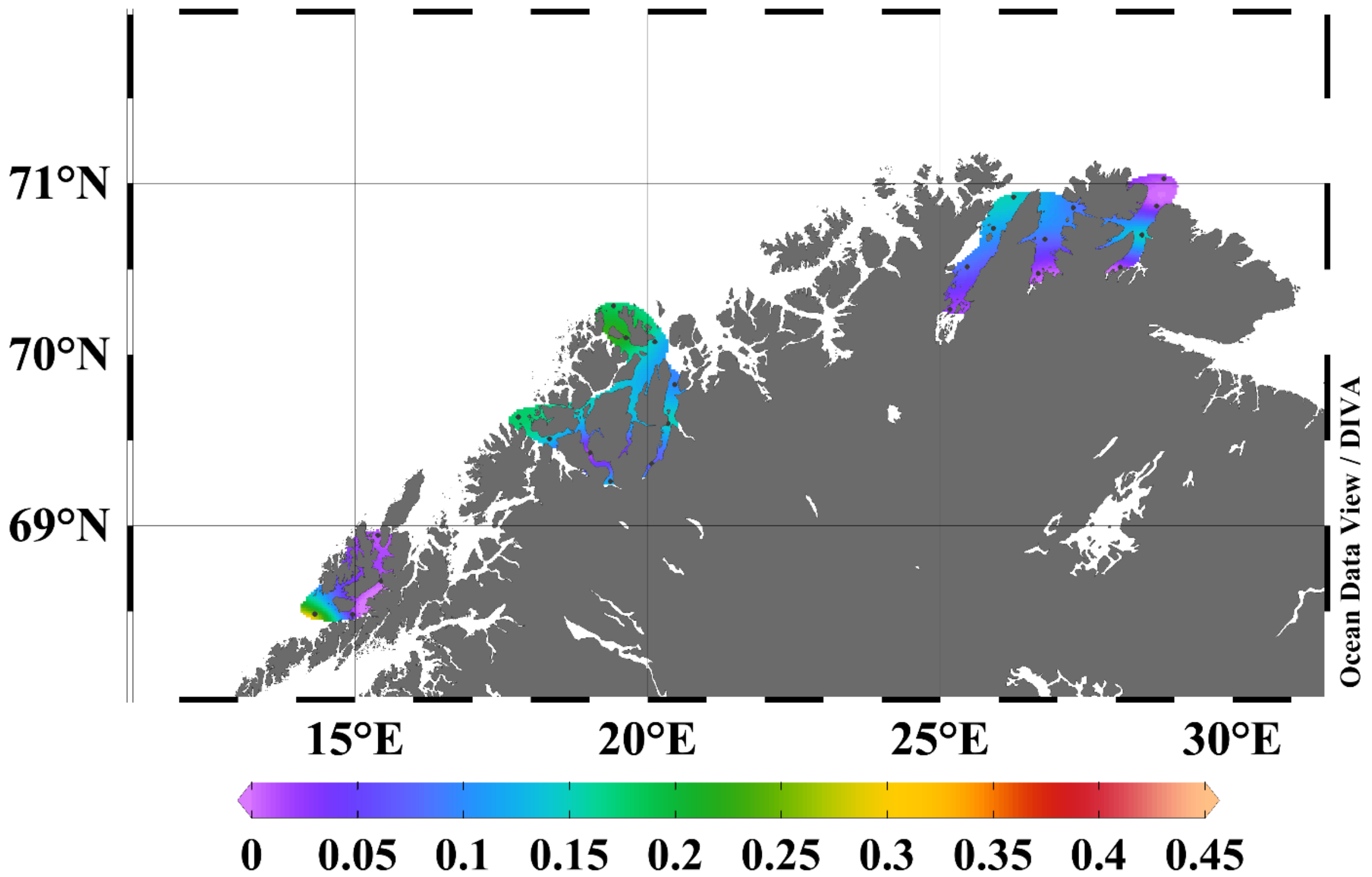

Figure S2. B) DCM NO_2 concentration

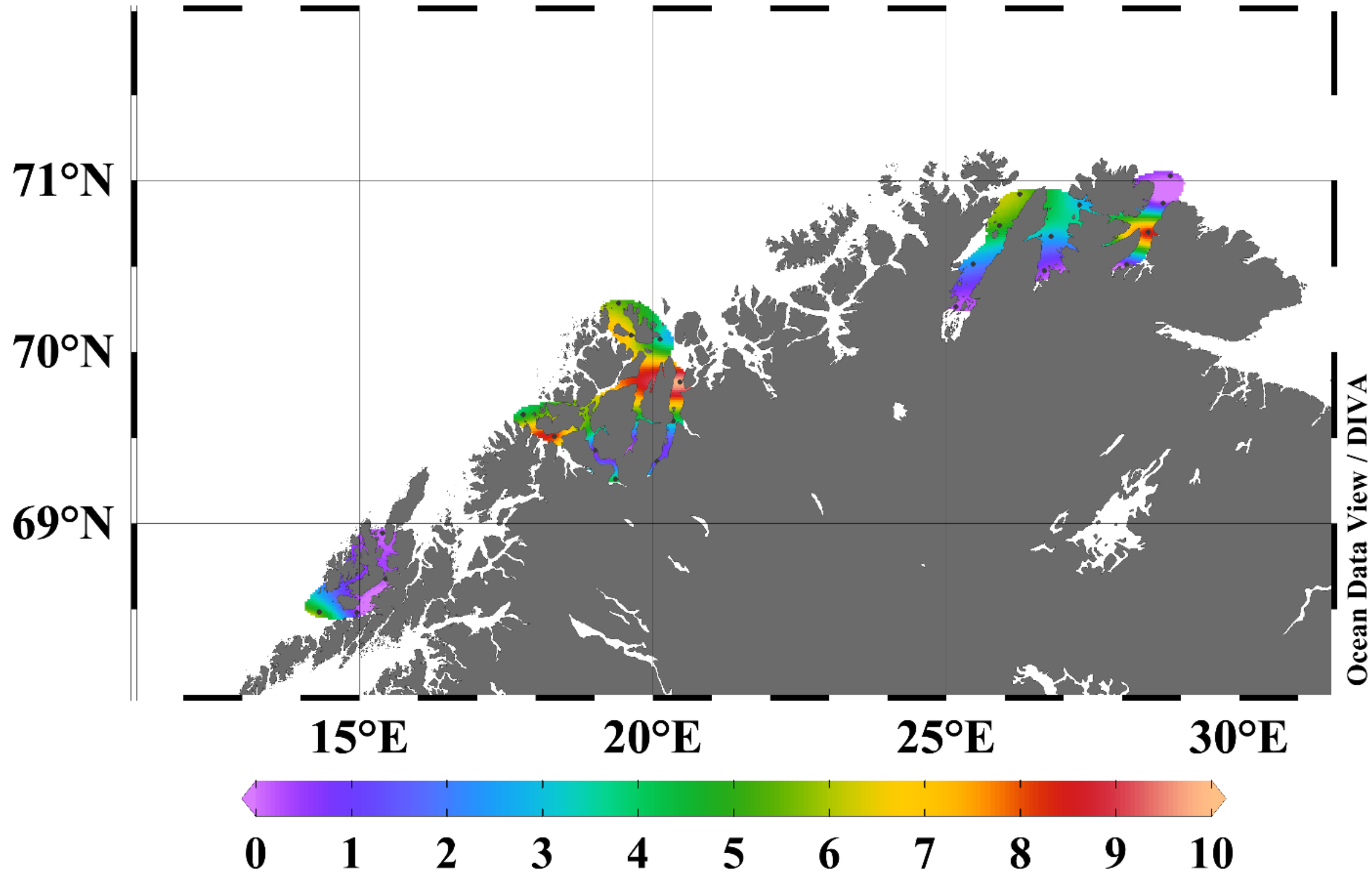

Figure S2. C) DCM NO_3 concentration

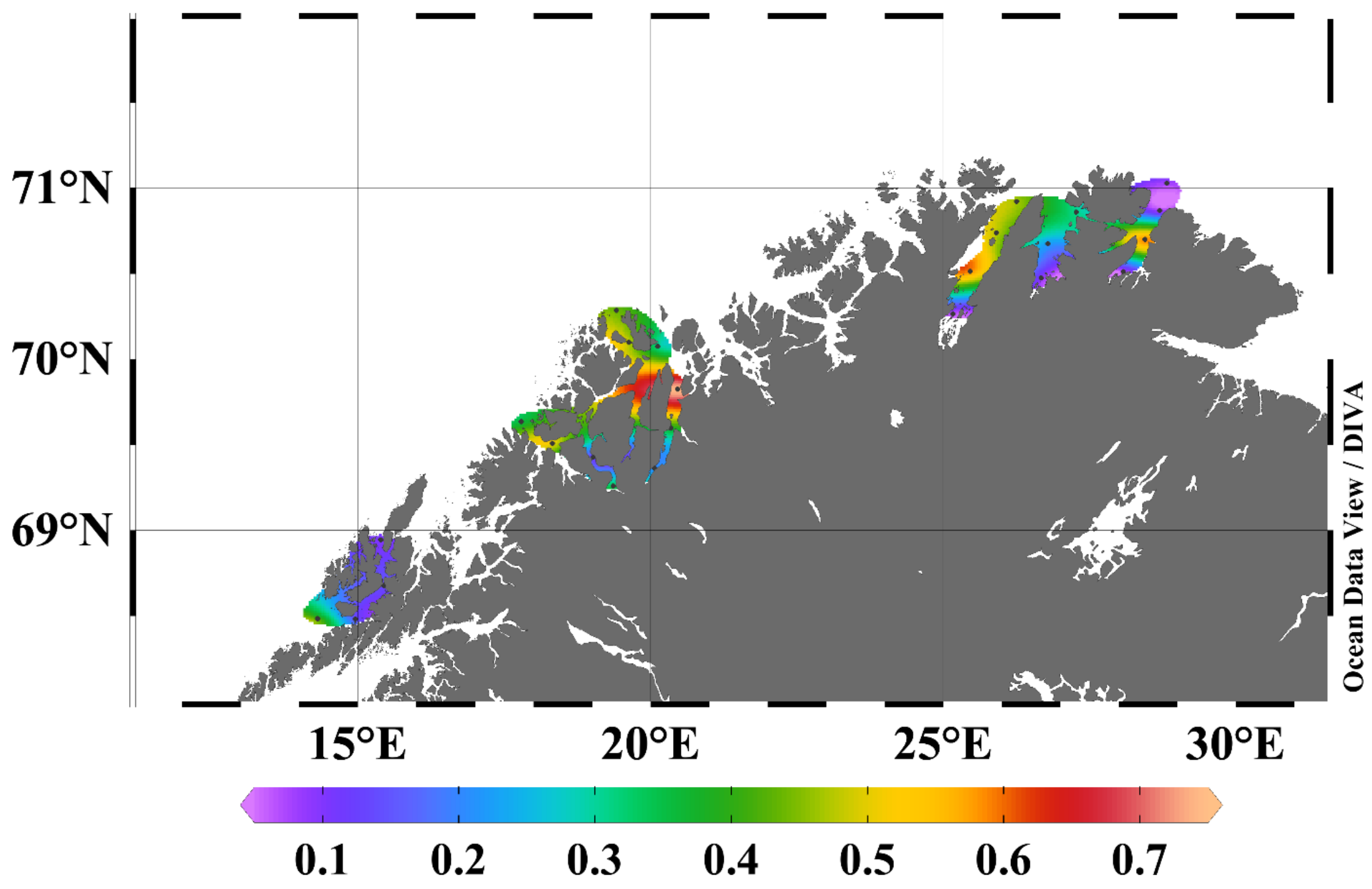

Figure S2. D) DCM PO_4 concentration

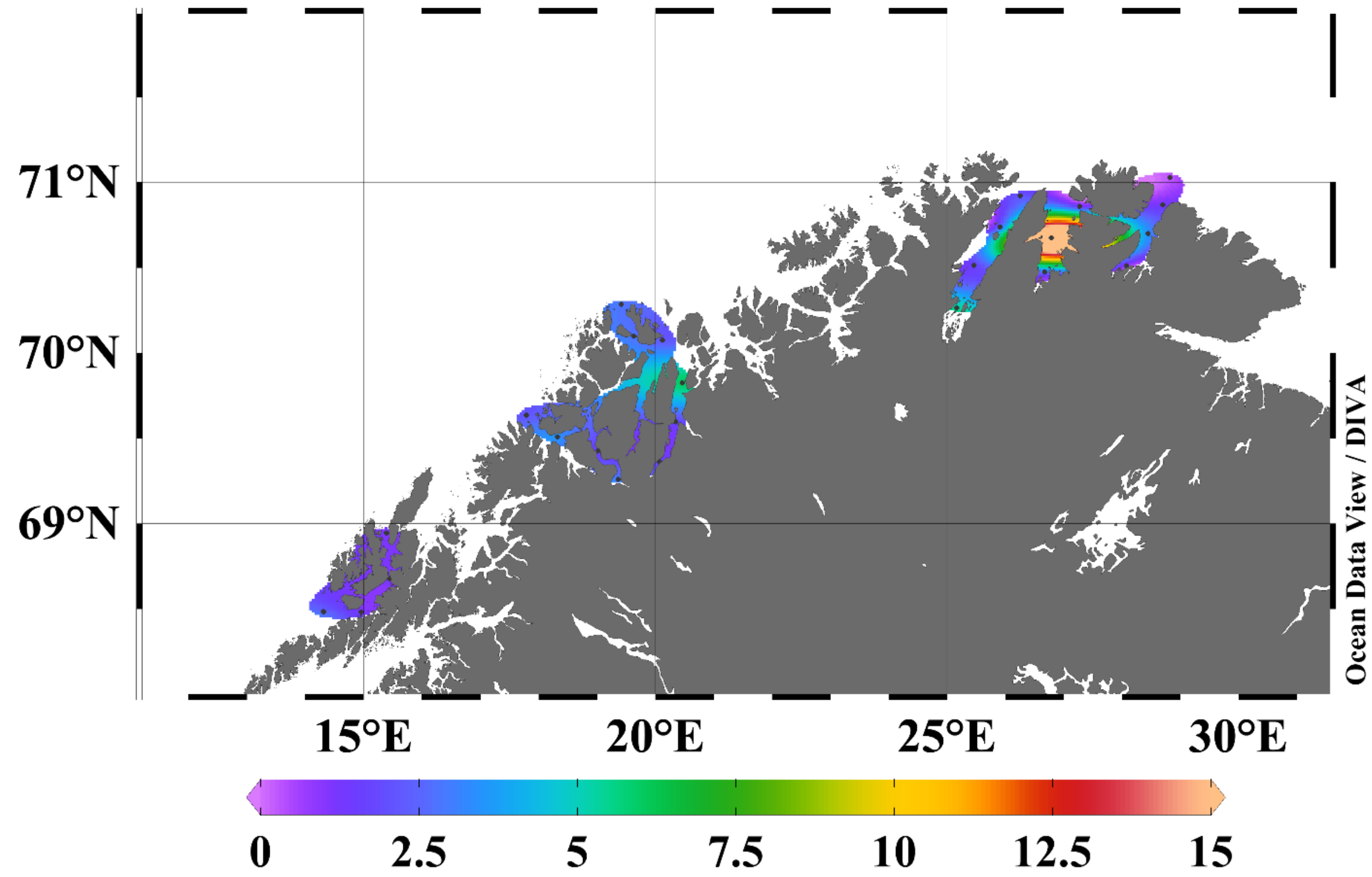

Figure S2. E) DCM SiO concentration

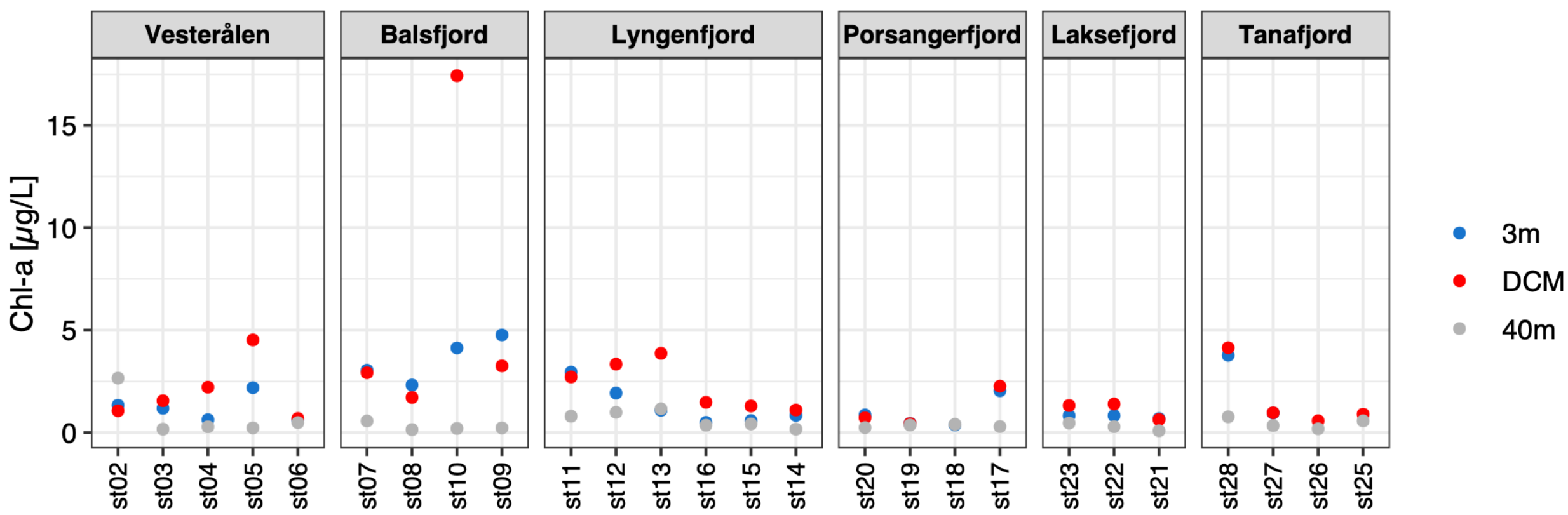

Figure S3. Chlorophyll-a distribution at each station and the 3 sampled depths.

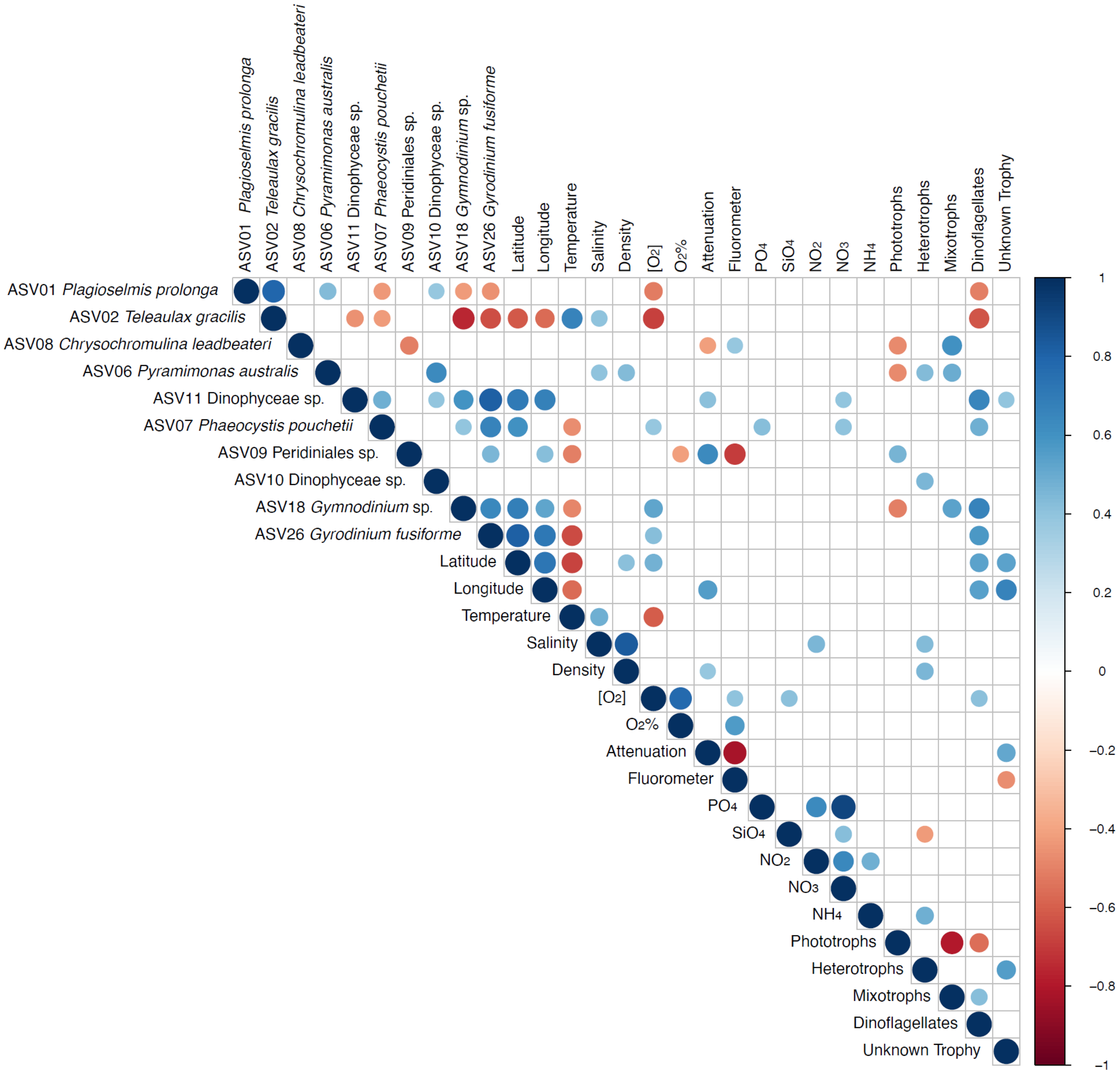

Figure S4. Spearman correlation matrix at p-values <0.05 between the most abundant ASVs, physicochemical parameters at the He533 cruise. The symbol size is inversely related to the p-value. Blue tones indicate positive correlations whereas red tones indicate negative ones.

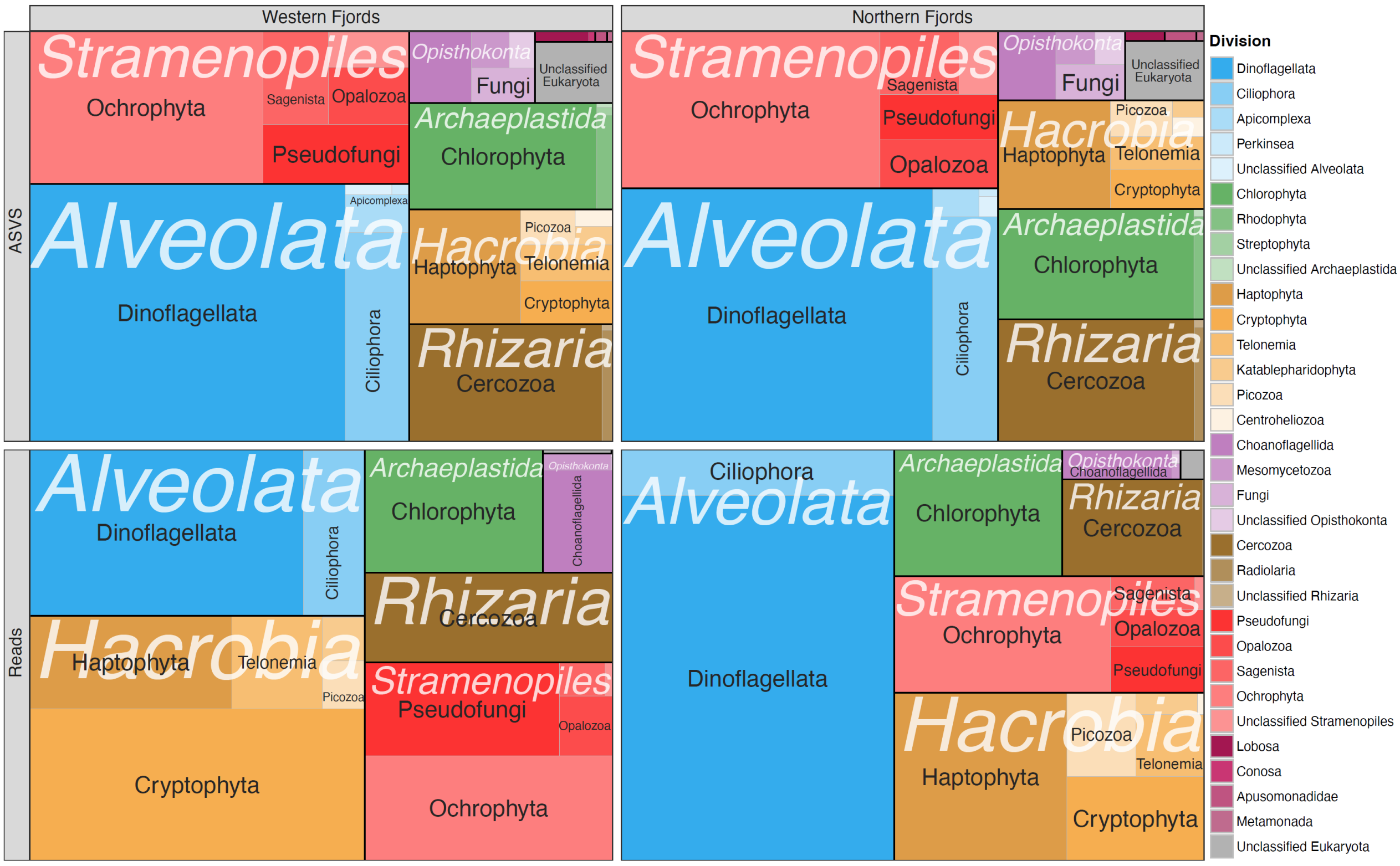

Figure S5. Treemaps displaying the Northern Norway fjords protist HTS community's taxonomic composition at supergroup and division levels: ASVs richness (above) and proportional read abundance (below).

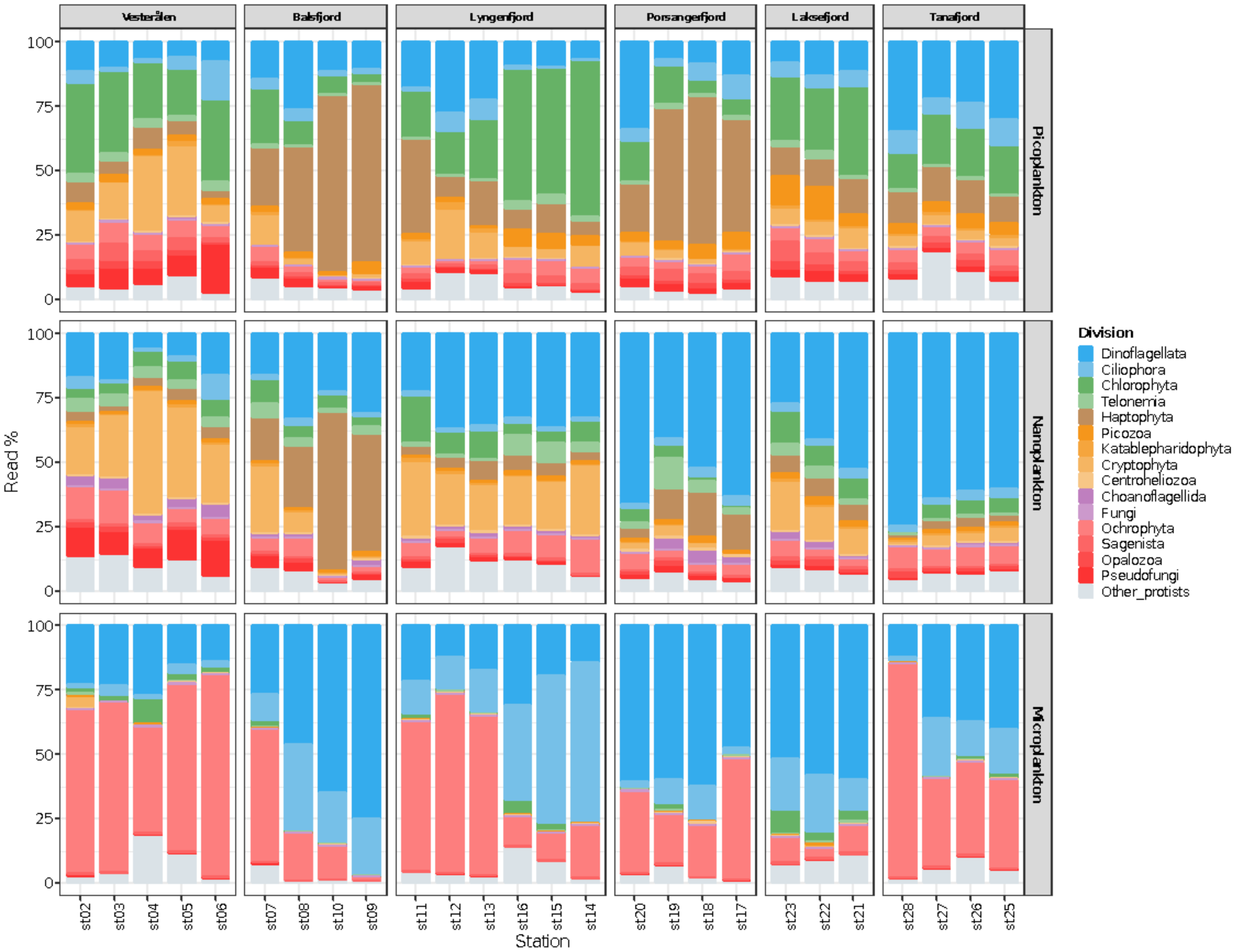

Figure S6. Taxonomic distribution of major taxonomic groups of protist divisions (as proportional read abundances) among the studied fjords by size fractions. Other protists include: Apicomplexa, Perkinsea, Rhodophyta, Streptophyta, Mesomycetozoa, Cercozoa, Radiolaria, Lobosa, Conosa, Apusomonadidae, Metamonada, and those that could not be classify further than to Alveolata, Archaeplastida, Opisthokonta, Rhizaria and Stramenopila. Stations are presented outer inwards the fjords.

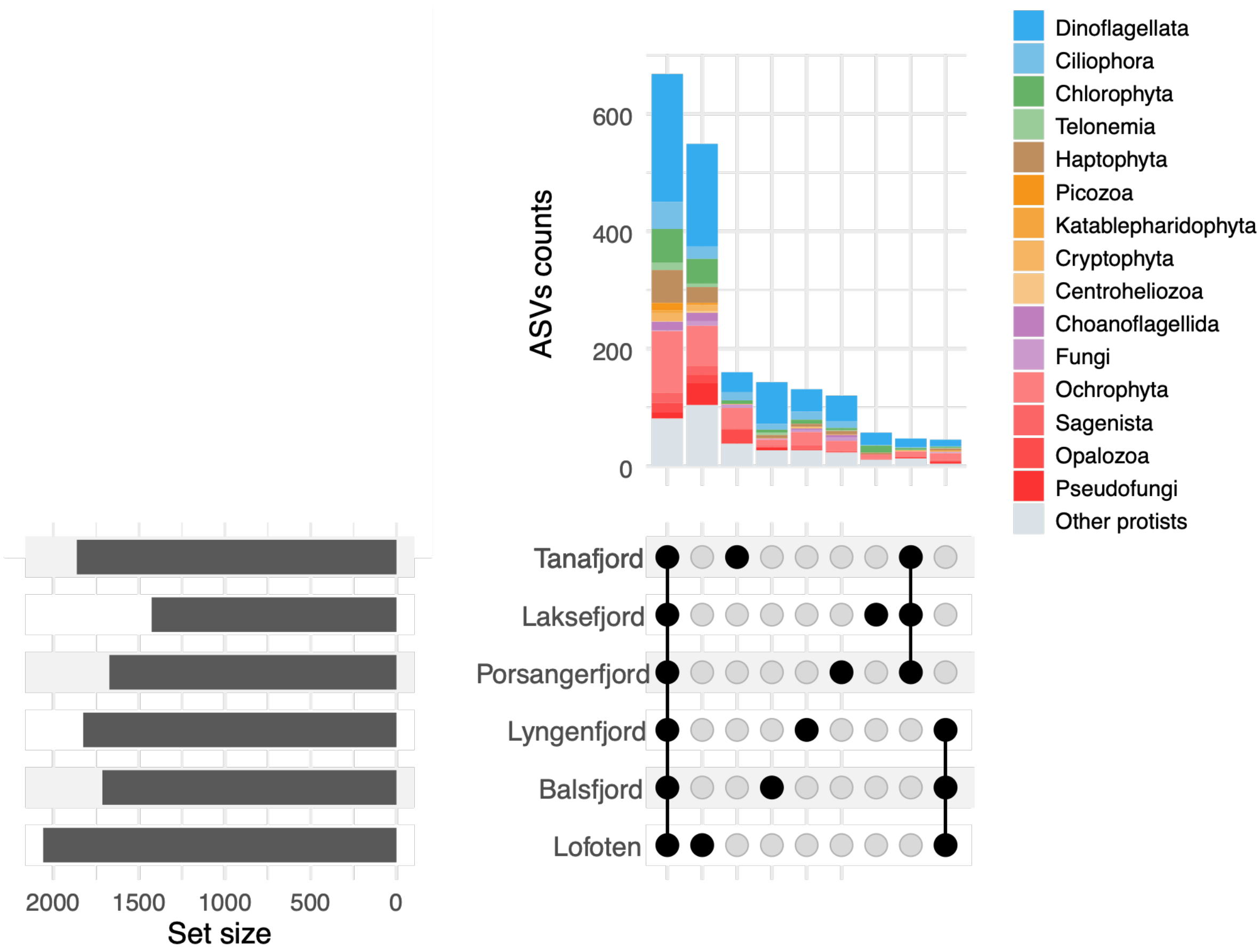

Figure S7. Distribution of protist groups ASVs across the five fjords and the Vesterålen area. Total number of Protist ASVs at each fjord or area is shown to the left (grey bars). The vertical bars indicate the number of ASVs unique to a particular fjord (single dark circles) or shared among fjords (dark circles connected by solid lines), as well as the taxa representation by color codes.

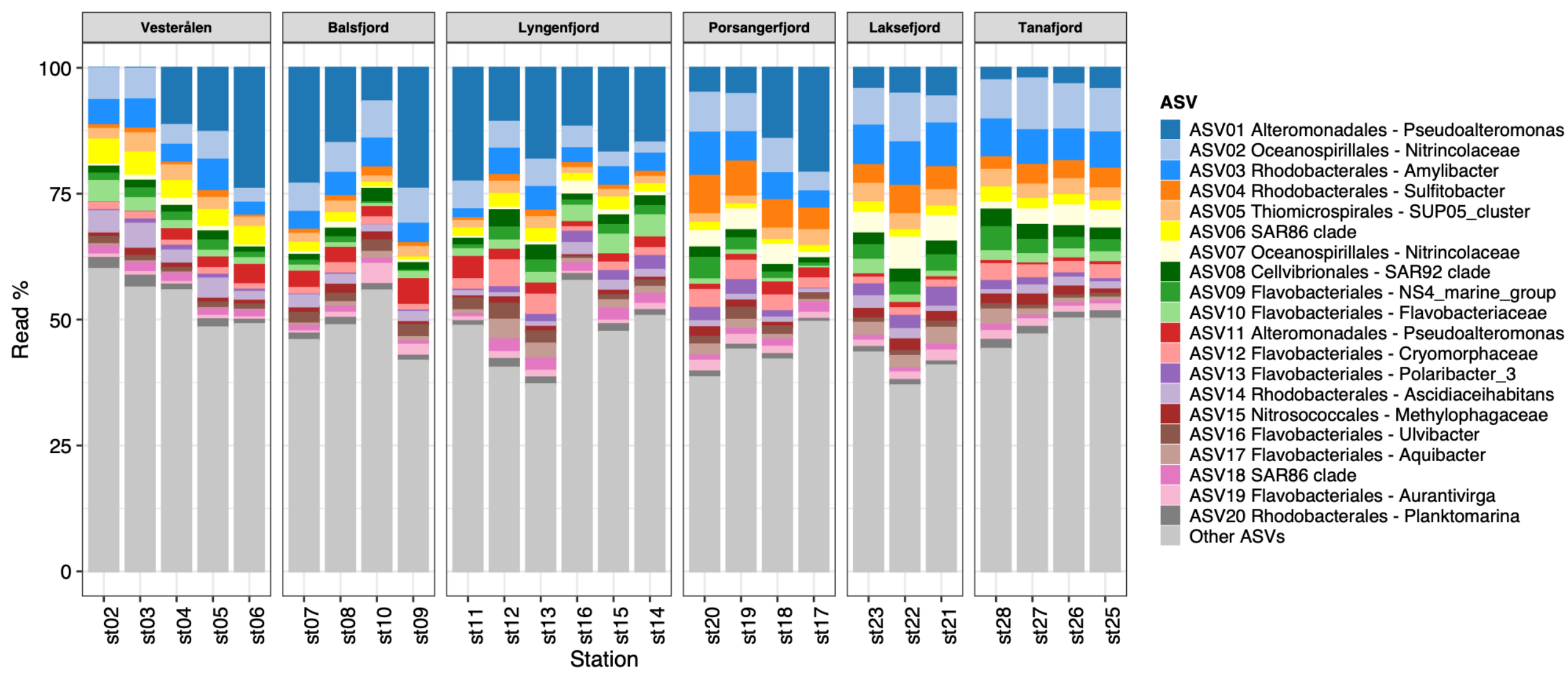

Figure S8. Taxonomic distribution of major taxonomic prokaryotic ASVs (as proportional read abundances) among the studied fjords by size fractions.
